# Supplementary material for: 1D axial heterostructure of hydrogen-bonded framework and metal-organic framework by metalation reaction
Source: Nat Commun. 2025 Nov 5;16:9768. doi: 10.1038/s41467-025-64715-1 (PMC12589647; doi:10.1038/s41467-025-64715-1)
Supplement: Supplementary file 1 — Supplementary Information [file 41467_2025_64715_MOESM1_ESM.pdf]

Supplementary Information

1D axial heterostructure of hydrogen-bonded framework and metal-organic framework by metalation reaction

Siquan Zhang[1], Yong-Sheng Wei[2*], Ellan K. Berdichevsky[1], Loris Lombardo[3], Zeyu Fan[1], Cheng Luo[1], Masahiko Tsujimoto[2], Nao Horike[2], Satoshi Horike[2,3,4*]

[1]Department of Synthetic Chemistry and Biological Chemistry, Graduate School of Engineering, Kyoto University, Kyoto 615-8510, Japan

[2]Institute for Integrated Cell-Material Sciences, Institute for Advanced Study, Kyoto University, Yoshida-Honmachi, Sakyo-ku, Kyoto 606-8501, Japan

[3]Department of Chemistry, Graduate School of Science, Kyoto University, Kitashirakawa-Oiwakecho, Kyoto 606-8502, Japan

[4]Department of Materials Science and Engineering, School of Molecular Science and Engineering, Vidyasirimedhi Institute of Science and Technology, Rayong 21210, Thailand

*E-mail: wei.yongsheng.3t@kyoto-u.ac.jp

*E-mail: horike.satoshi.3r@kyoto-u.ac.jp

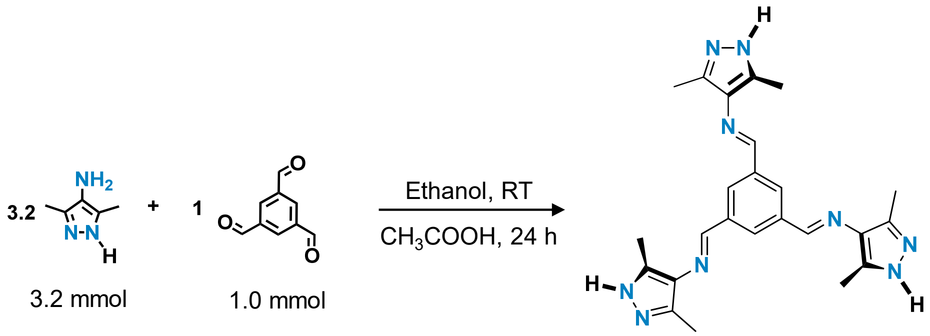

Supplementary Fig. 1. Synthesis of **HOF-a** (**H_3L1**).

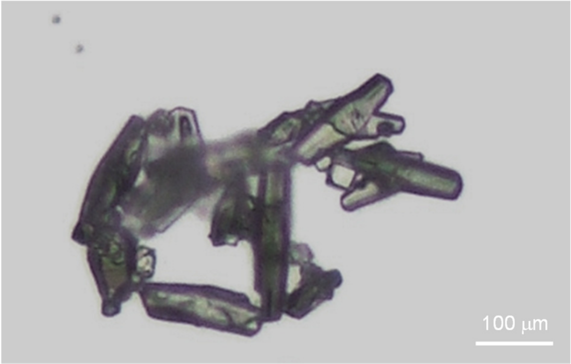

Supplementary Fig. 2. The optical image of **HOF-a**.

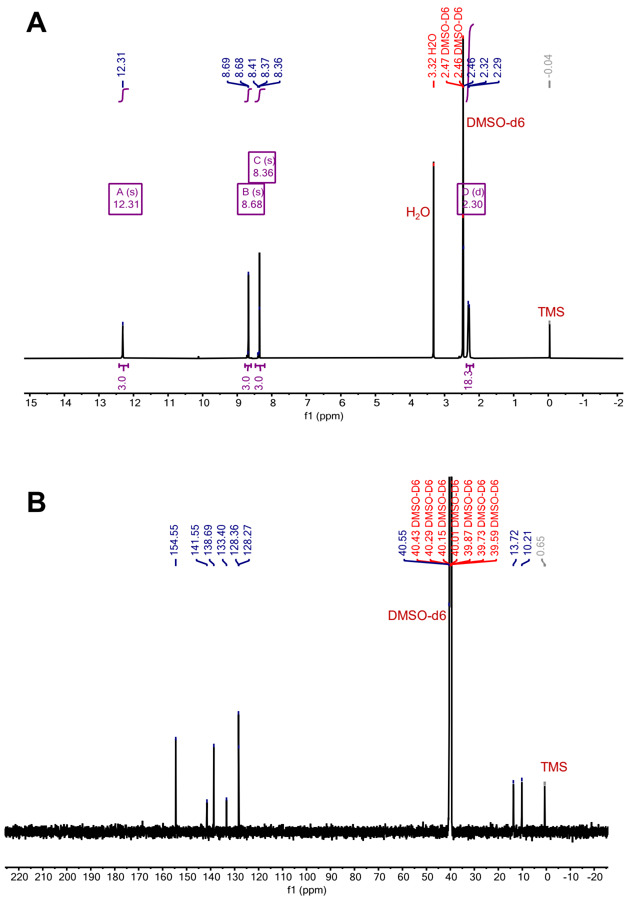

Supplementary Fig. 3. (A) ^{1}H NMR and (B) ^{13}C NMR of **H_3L1** (**HOF-a**).

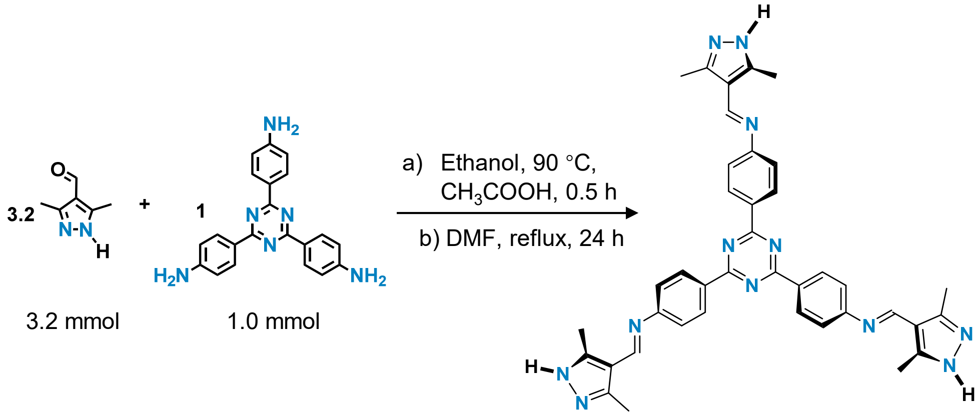

Supplementary Fig. 4. Synthesis of **HOF-b** (**H_3L2**).

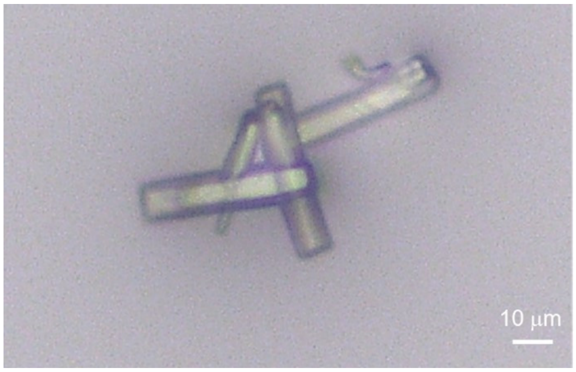

Supplementary Fig. 5. The optical image of **HOF-b**.

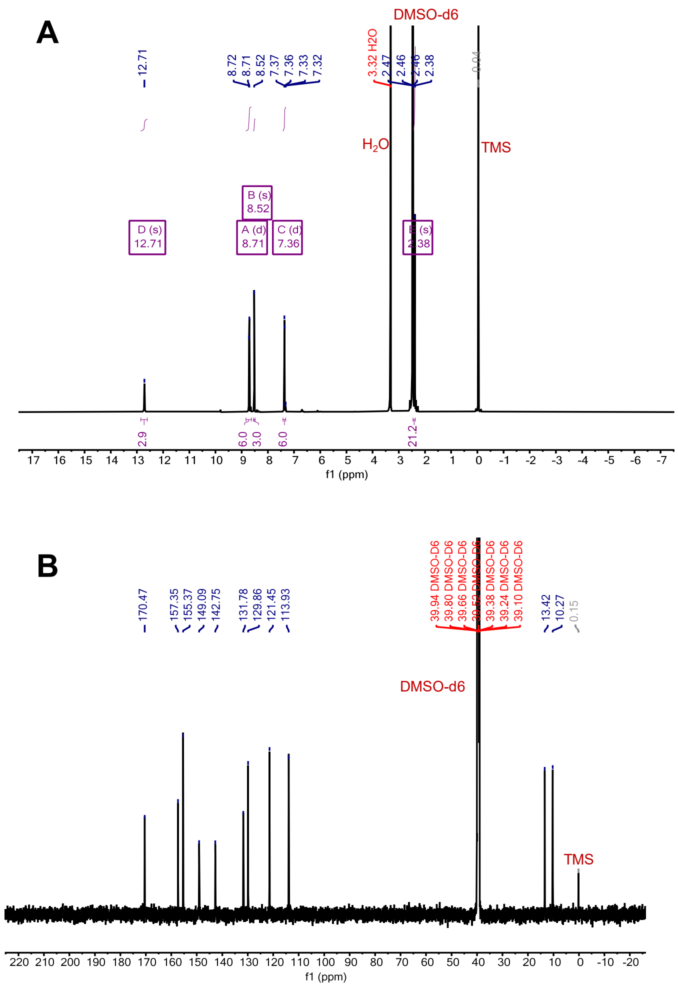

Supplementary Fig. 6. (A) ^{1}H NMR and (B) ^{13}C NMR of **H_3L2** (**HOF-b**).

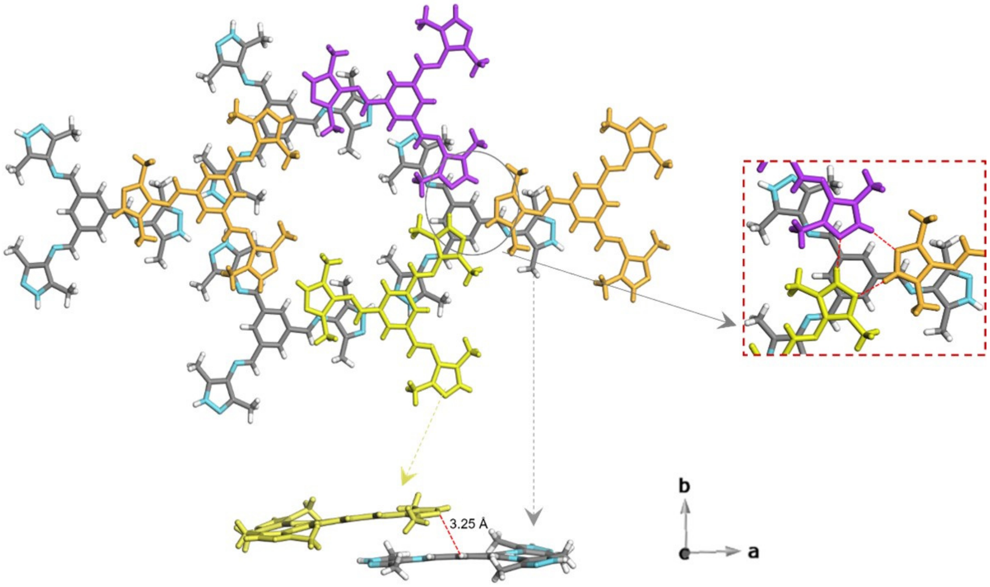

Supplementary Fig. 7. Crystal structure of **HOF-a**. Atom colors: C, gray; N, blue; H, white.

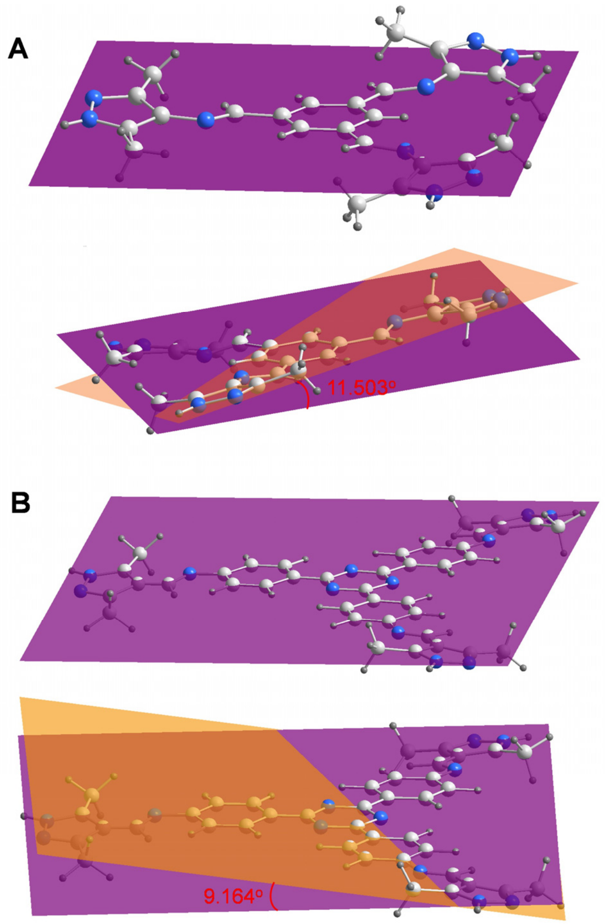

Supplementary Fig. 8. The non-coplanar structures in (A) **HOF-a** and (B) **HOF-b**. Atom colors: C, white; N, blue; H, dark gray.

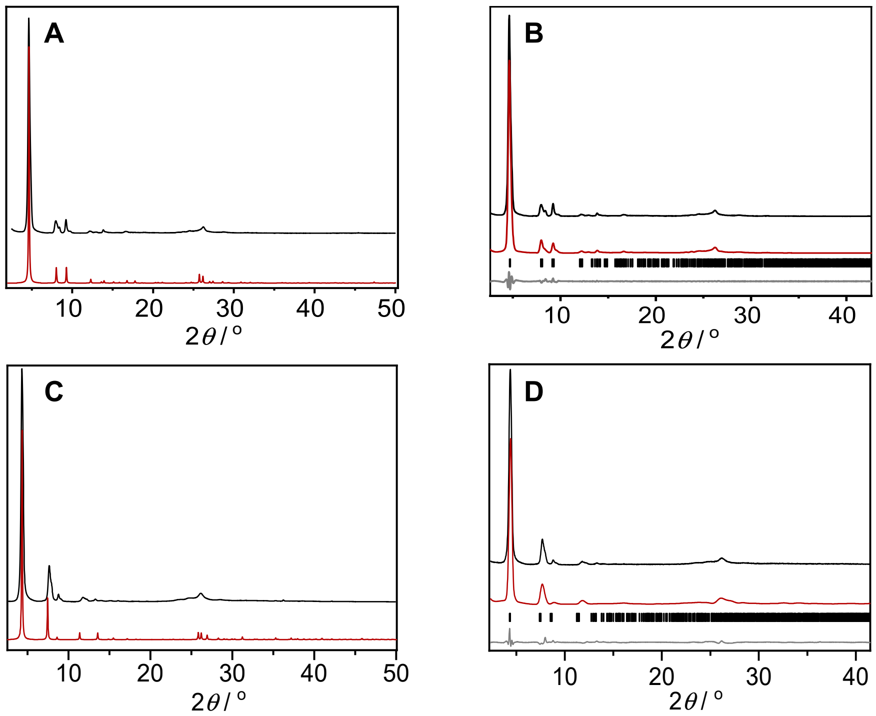

Supplementary Fig. 9. (A) Experimental (black curve) and simulated (red curve) PXRD patterns of **HOF-b**. (B) Experimental (black curve), refined (red curve) PXRD patterns, Bragg positions (black dots), and the difference (gray curve) of **HOF-b** (R_w = 5.64%, R_{wp} = 8.08%). (C) Experimental (black curve) and simulated (red curve) PXRD patterns of **MOF-b-Cu**. (D) Experimental (black curve), refined (red curve) PXRD patterns, Bragg positions (black dots), and the difference (gray curve) of **MOF-b-Cu** (R_w = 2.71%, R_{wp} = 5.32%).

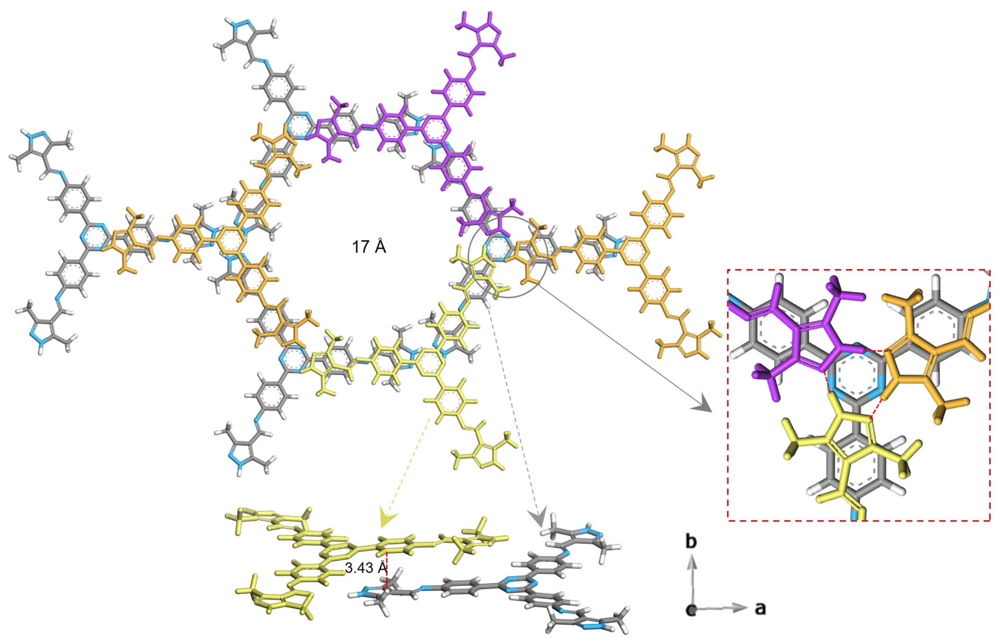

Supplementary Fig. 10. Crystal structure of **HOF-b**. Atom colors: C, gray; N, blue; H, white.

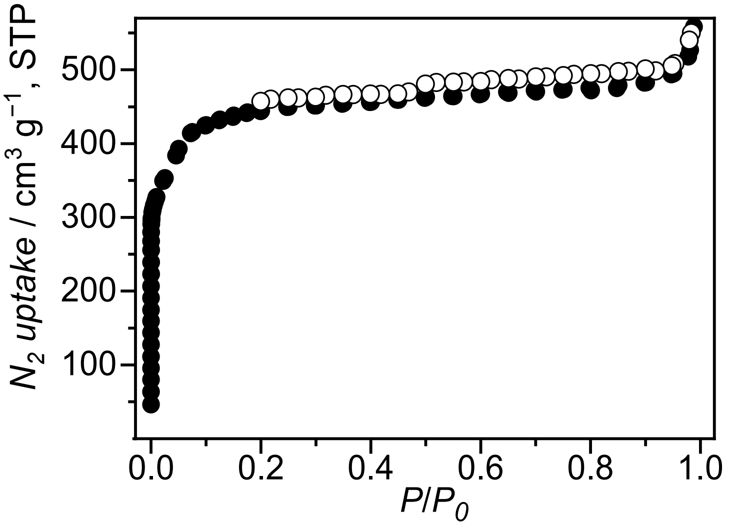

Supplementary Fig. 11. N_2 adsorption (●) and desorption (○) isotherms of **HOF-a** at 77 K.

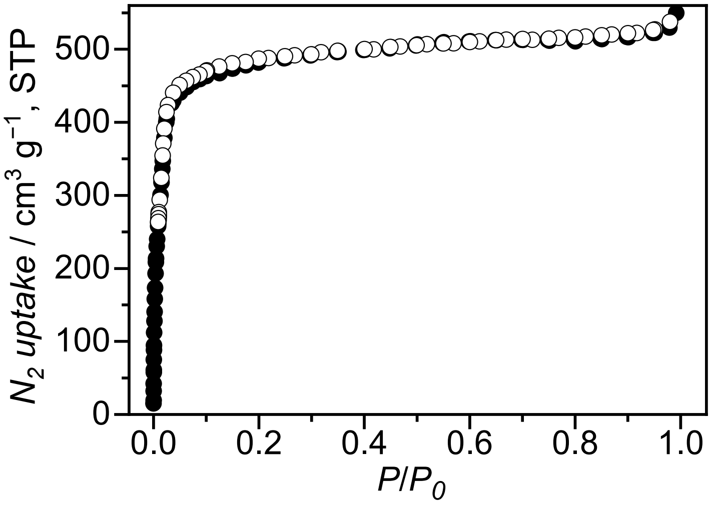

Supplementary Fig. 12. N_2 adsorption (●) and desorption (○) isotherms of **HOF-b** at 77 K.

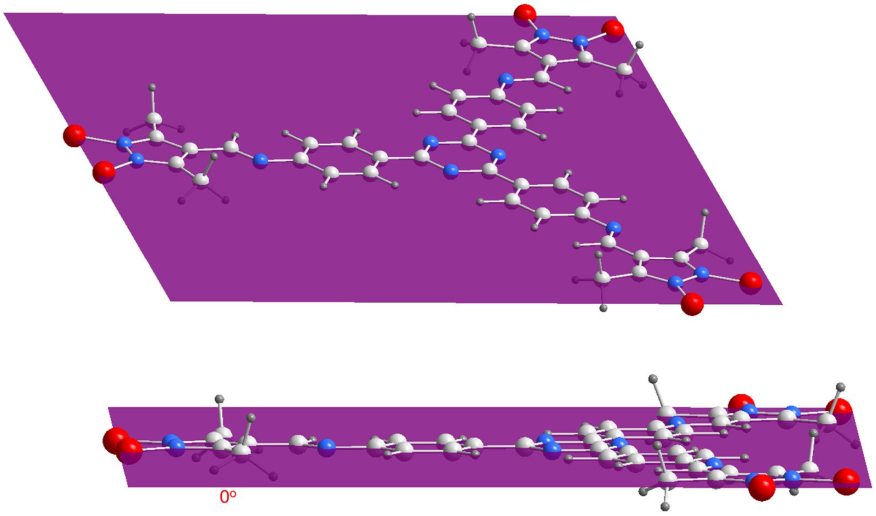

Supplementary Fig. 13. The planar structure in **MOF-b-Cu**. Atom colors: C, white; N, blue; H, dark gray; Cu, red.

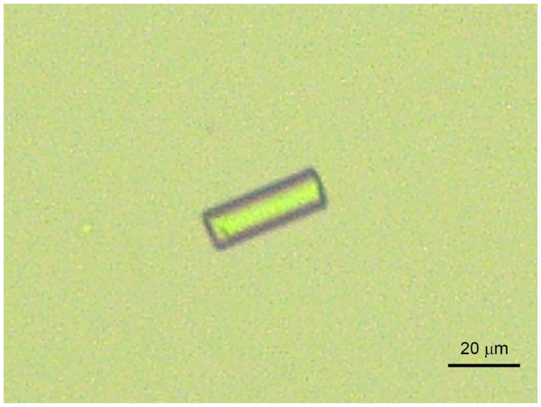

Supplementary Fig. 14. Optical image of **MOF-b-Cu**.

Supplementary Table 1. Synthesis conditions and product characteristics.[a]

Entry	Method	**HOF-b** / mmol	Copper salts / mmol	Catalyst	Solvent (50 mL)	Crystallinity
1	Direct syntheses	0.1	0.6 $Cu(CF_3SO_3)$		*N,N*-dimethylacetamide	amorphous
2		0.1	0.6 $Cu(CF_3SO_3)$		dimethyl sulfoxide	amorphous
3		0.1	0.6 $Cu(CF_3SO_3)$		DMF	amorphous
4		0.1	0.6 $Cu(CF_3SO_3)$	CH_3COOH 200 μL	DMF	amorphous
5		0.1	0.6 $Cu(CF_3SO_3)$		DMF/methanol 3/1	amorphous
6		0.1	0.6 $Cu(CF_3SO_3)$		DMF/methanol 3/2	amorphous
7		0.1	0.6 $Cu(CF_3SO_3)$		DMF/methanol 3/4	amorphous
8	Metalation	0.1	0.6 $Cu(CF_3SO_3)$		methanol	crystalline (**MOF-b-Cu**)
9		0.1	0.6 $Cu(CF_3SO_3)$		ethanol	crystalline
10		0.1	0.6 $Cu(CF_3SO_3)$		acetone	crystalline
11		0.1	0.6 $Cu(CF_3SO_3)$		*o*-dichlorobenzene	crystalline
12		0.1	0.6 $Cu(CF_3SO_3)$		1,4-dioxane	crystalline
13		0.1	0.6 $Cu(CF_3SO_3)$		acetonitrile	crystalline
14		0.1	0.6 $Cu(SO_4)$		methanol	amorphous
15		0.1	0.6 $Cu(CF_3SO_3)_2$		methanol	amorphous
16		0.1	0.6 $Cu(NO_3)_2$		methanol	amorphous
17		0.1	0.6		methanol	crystalline

			$Cu(BF_4)$			
18		0.1	0.6 $Cu(PF_6)$		methanol	crystalline
19		0.1	0.6 $Cu(CF_3SO_3)$	CH_3COOH 200 μL	methanol	crystalline
20		0.1	0.6 $Cu(CF_3SO_3)$	HBF_4 200 μL	methanol	crystalline
21		0.1	0.6 $Cu(CF_3SO_3)$	CF_3COOH 200 μL	methanol	crystalline
22		0.1	0.6 $Cu(CF_3SO_3)$	CF_3SO_3H 200 μL	methanol	crystalline

[a]All the entries were performed at 298 K for two days.

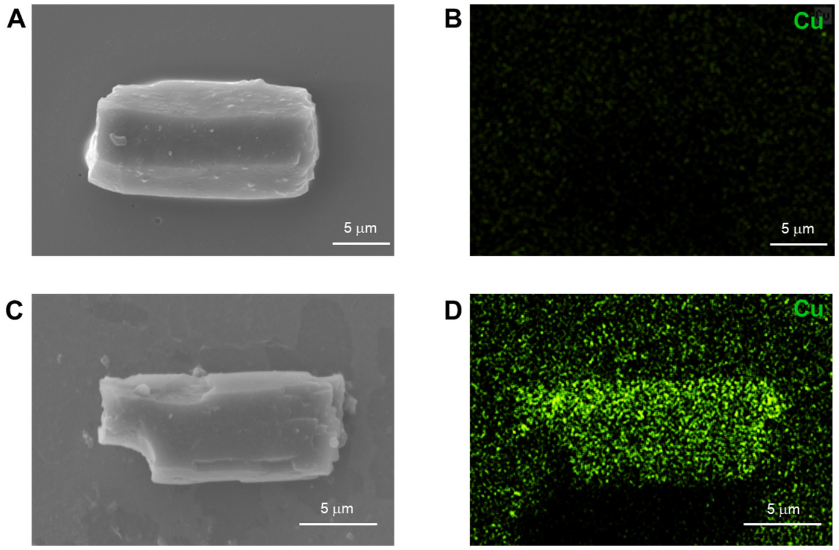

Supplementary Fig. 15. (A) SEM and (B) EDS mapping images of **HOF-b**. (C) SEM and (D) EDS mapping images of **MOF-b-Cu**.

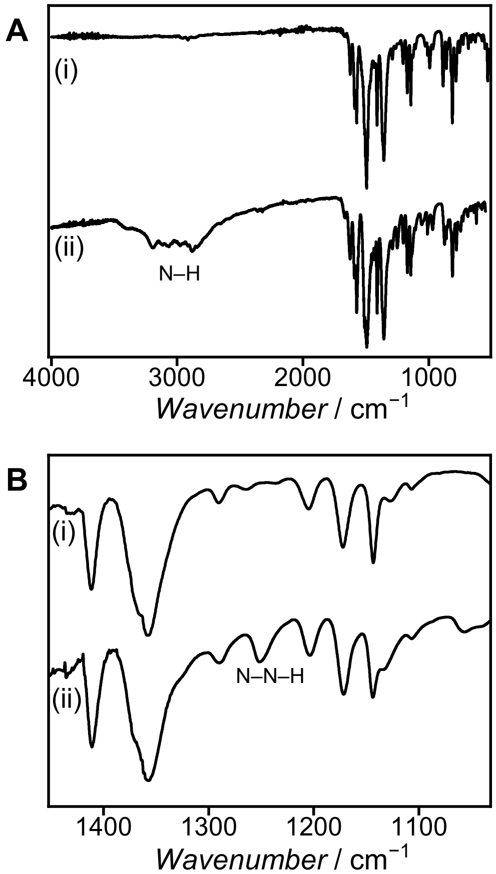

Supplementary Fig. 16. FT-IR spectra of (i) **MOF-b-Cu** and (ii) **HOF-b** in the wavenumber of (A) 4000 to 600 cm^{-1} and (B) 1450 to 1050 cm^{-1}.

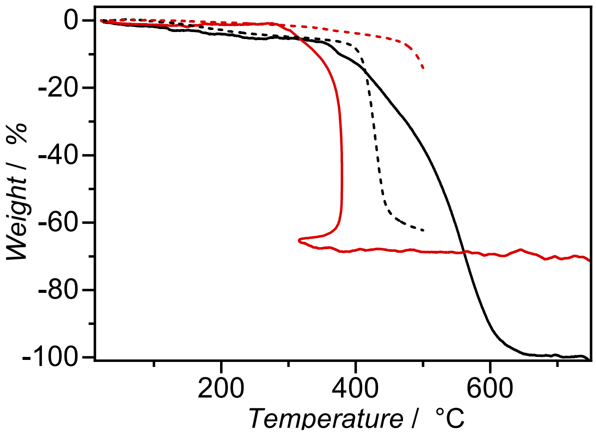

Supplementary Fig. 17. TGA profiles of (A) **HOF-b** in air (black solid curve), (B) **HOF-b** in Ar (black dashed curve), (C) **MOF-b-Cu** in air (red solid curve), and (D) **MOF-b-Cu** in Ar (red dashed curve). The irregularity observed in the TGA profile of **MOF-b-Cu** in air at the temperature of 318 to 383 °C is due to its abrupt decomposition with an intense exothermic reaction[1–4].

Supplementary Table 2. The metalation conversion calculated[5, 6] by TGA profiles in air of **HOF|MOF-*x*h** (x = 0, 0.5, 2.5, 6, 10, 20, and 48), **MOF-b-Ag**, and **MOF-b-Au**.

Samples	Weight loss at 200 °C (wt%)	Weight loss at 500 °C (wt%)	Metal content (wt%)	Theoretical ($C_{13}H_{11}MN_4$) (wt%)	Conv.
**HOF-b**			0	0	0
**HOF|MOF-0.5h**	3.8%	96%	3.3%	22.1%	14.9%
**HOF|MOF-2.5h**	1.4%	94.6%	4.4%	22.1%	19.9%
**HOF|MOF-6h**	2.0%	85.4%	11.9%	22.1%	53.8%
**HOF|MOF-10h**	8.7%	84.3%	13.8%	22.1%	62.4%
**HOF|MOF-20h**	4.8%	80.5%	16.4%	22.1%	74.3%
**MOF-b-Cu**	1.2%	72.6%	22.1%	22.1%	~100%
**MOF-b-Ag**	2.7%	70.2%	30.6%	32.5%	94.2%

**MOF-b-Au**	0.8%	56.5%	43.6%	46.8%	93.2%

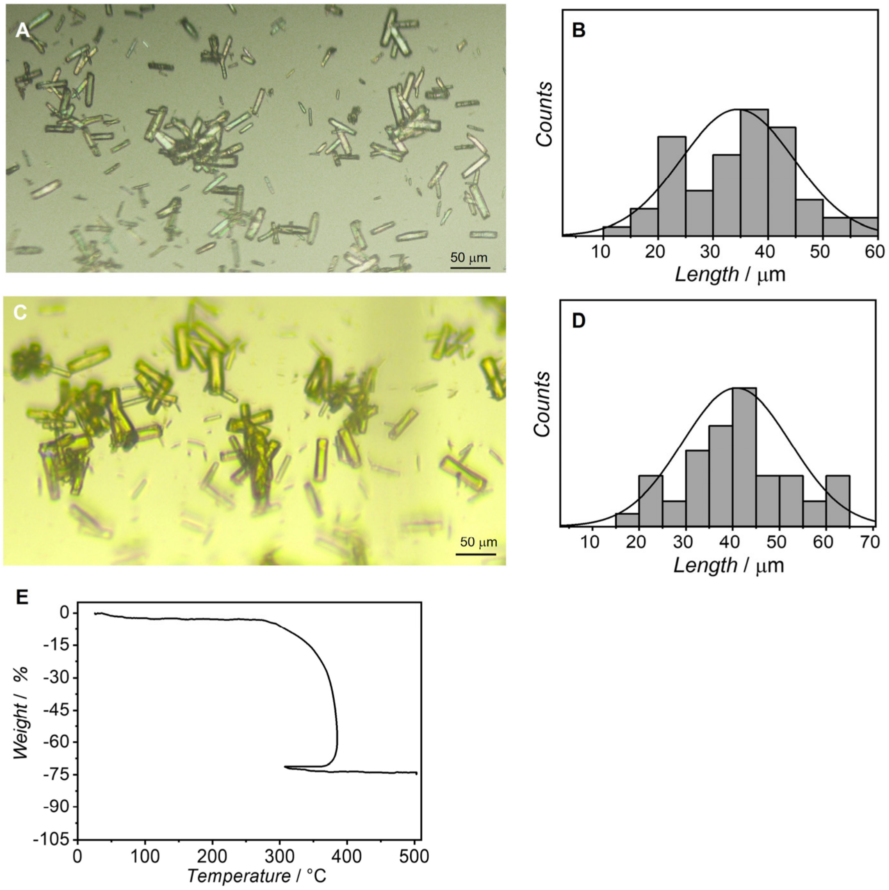

Supplementary Fig. 18. (A) The optical images of **HOF-b** crystals. (B) Normal distribution of **HOF-b** crystal sizes measured from (A). (C) The optical images of **HOF-b** crystals. (D) Normal distribution of **HOF-b** crystal sizes measured from (C). (E) TGA profiles in air of **MOF-b-Cu** (60 μm).

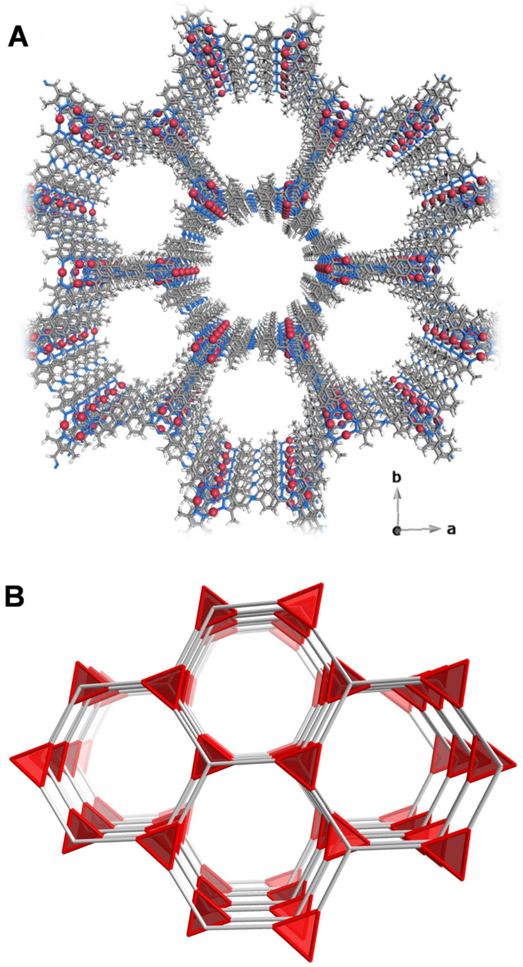

Supplementary Fig. 19. (A) Crystal structure from **MOF-b-Cu** and (B) the **hnb** net of **MOF-b-Cu**. Atom colors: C, gray; N, blue; H, white; Cu, red.

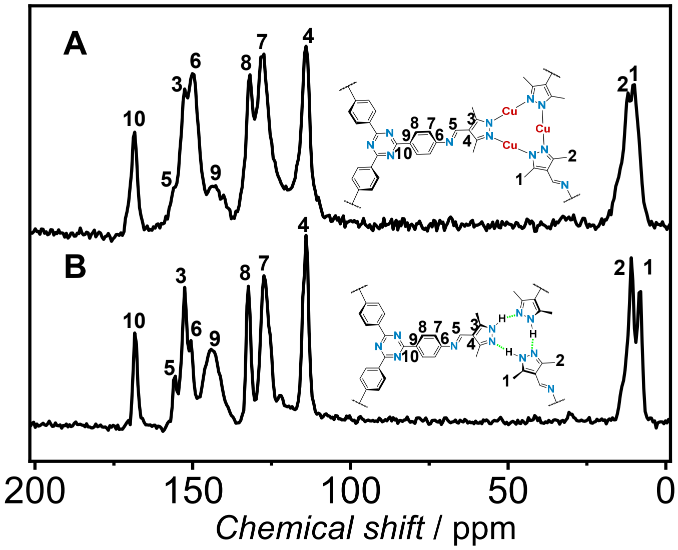

Supplementary Fig. 20. Solid-state ^{13}C NMR spectra of (A) **MOF-b-Cu** and (B) **HOF-b** and assignments of peaks[7, 8].

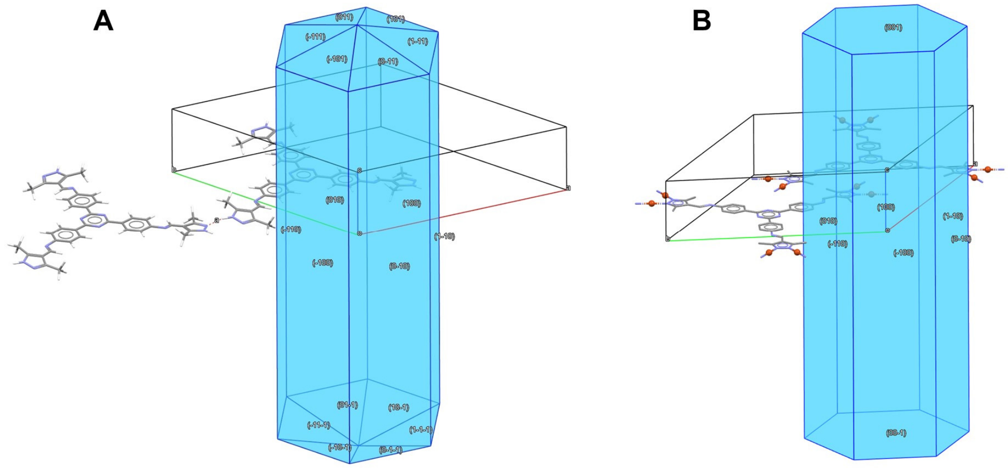

Supplementary Fig. 21. Tridimensional BFDH theoretical crystal morphologies of (A) **HOF-b** and (B) **MOF-b-Cu** drawn out by Mercury software[9, 10]. Atom colors: C, gray; N, light purple; H, white; Cu, red.

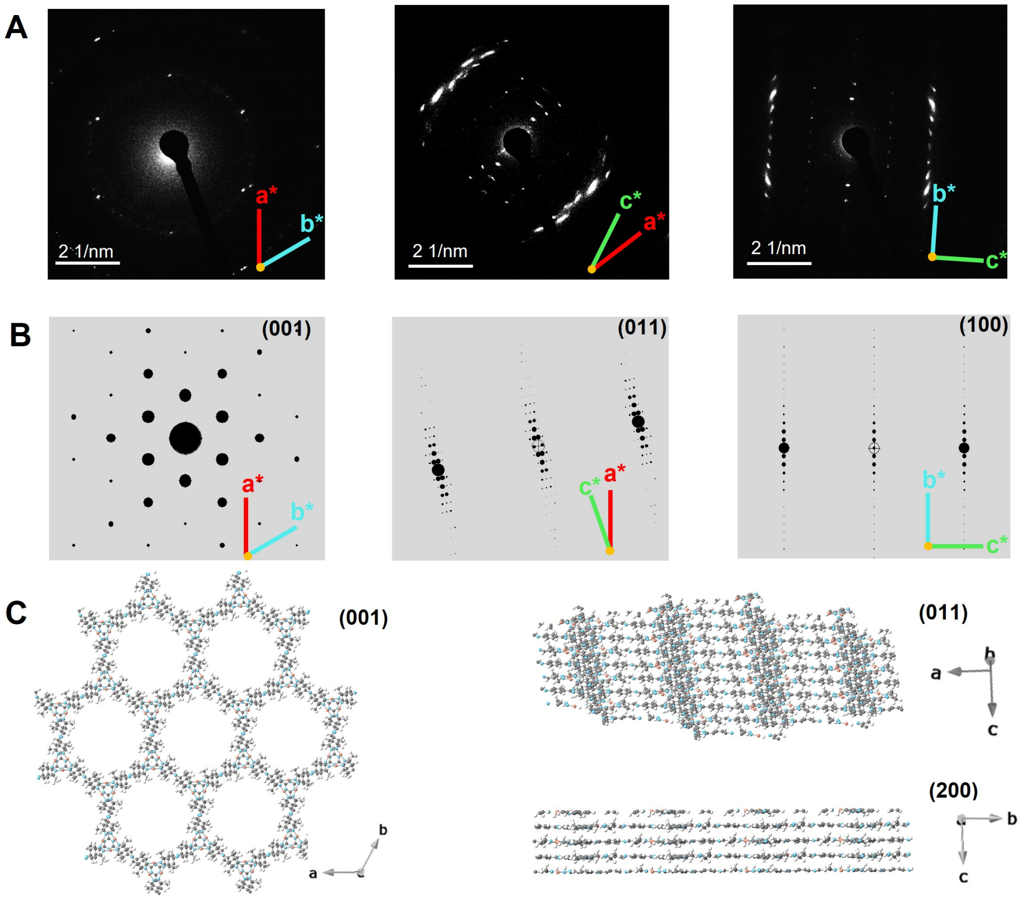

Supplementary Fig. 22. (A) SAED, (B) simulated diffraction patterns, and (C) their corresponding facets of **MOF-b-Cu**. Atom colors: C, gray; N, blue; H, white; Cu, red.

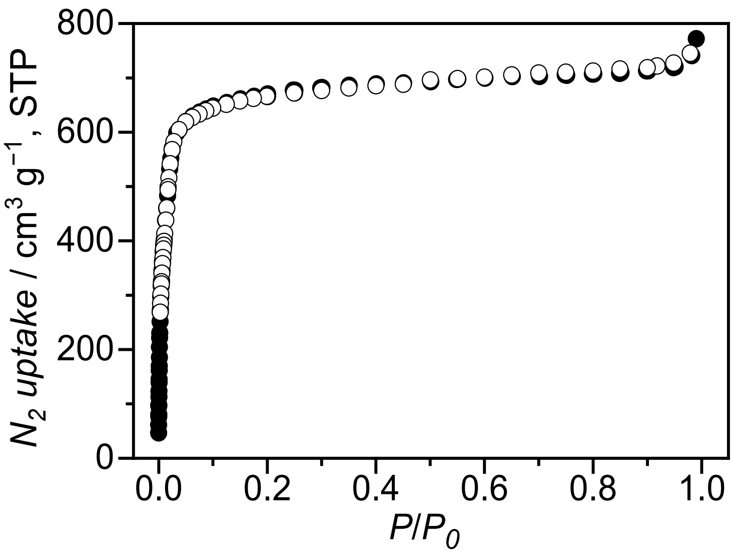

Supplementary Fig. 23. N_2 adsorption (●) and desorption (○) isotherms of **MOF-b-Cu** at 77 K.

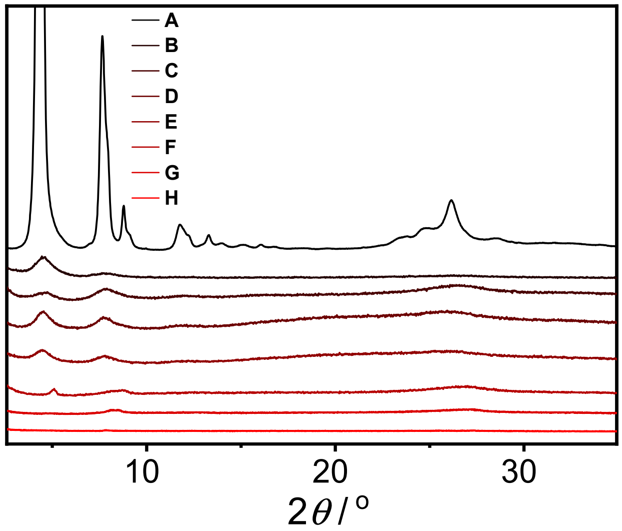

Supplementary Fig. 24. PXRD patterns of (A) **MOF-b-Cu**, samples from direct synthesis of (B) entry 1, (C) entry 2, (D) entry 3, (E) entry 4, (F) entry 5, (G) entry 6, and (H) entry 7.

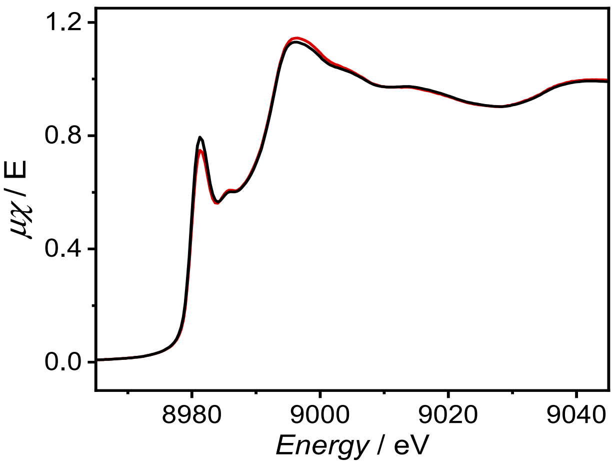

Supplementary Fig. 25. Cu *K*-edge X-ray absorption near edge structure (XANES) spectra of (A) the sample from direct synthesis of entry 3 (red curve) and (B) **MOF-b-Cu** (black curve).

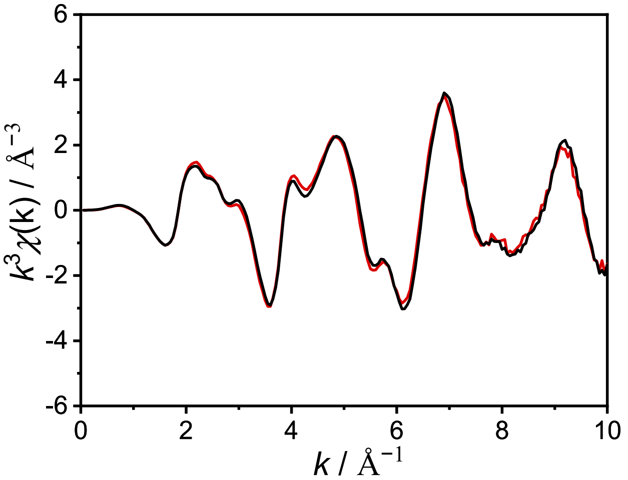

Supplementary Fig. 26. *k* space of (A) the sample from direct synthesis of entry 3 (red curve) and (B) **MOF-b-Cu** (black curve).

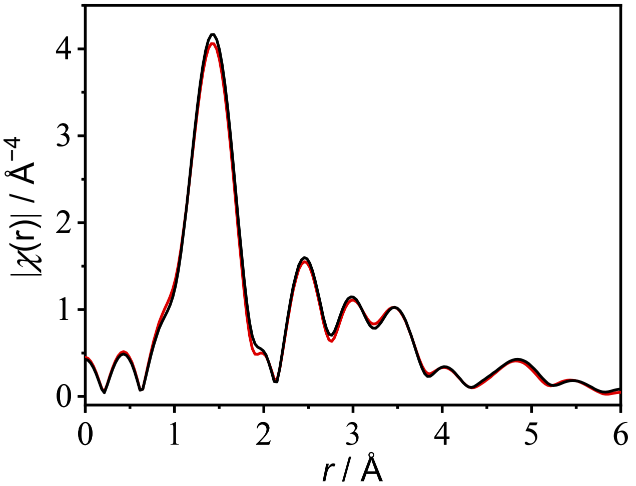

Figure 27. Cu *K*-edge k^3-weighted EXAFS spectra in *R* space of (A) the sample from direct synthesis of entry 3 (red curve) and (B) **MOF-b-Cu** (black curve).

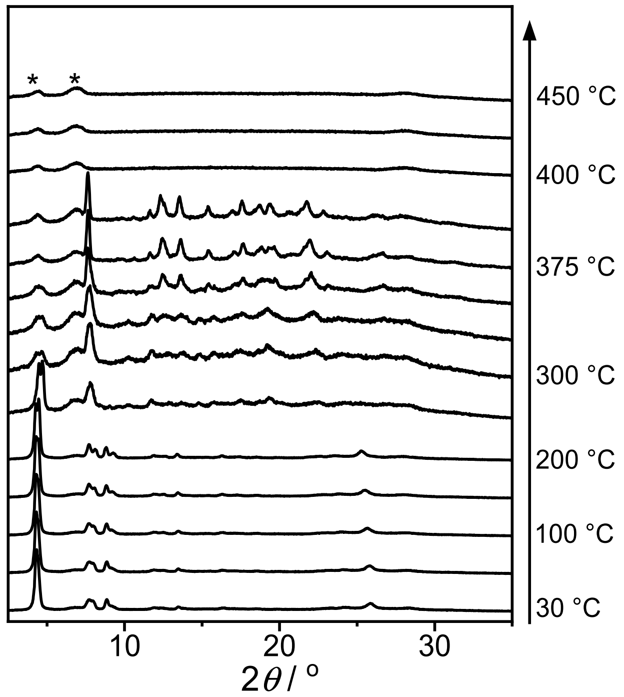

Supplementary Fig. 28. VT-PXRD patterns of **HOF-b**. Asterisks are background.

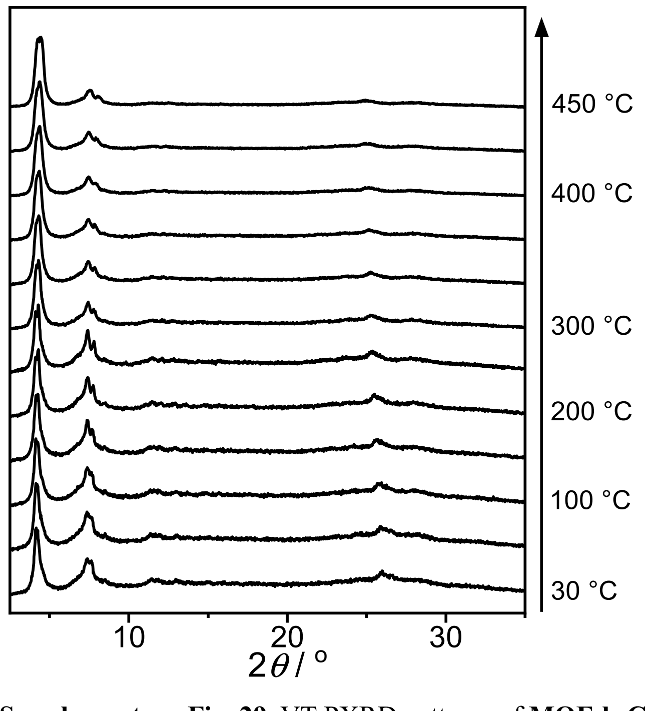

Supplementary Fig. 29. VT-PXRD patterns of **MOF-b-Cu**.

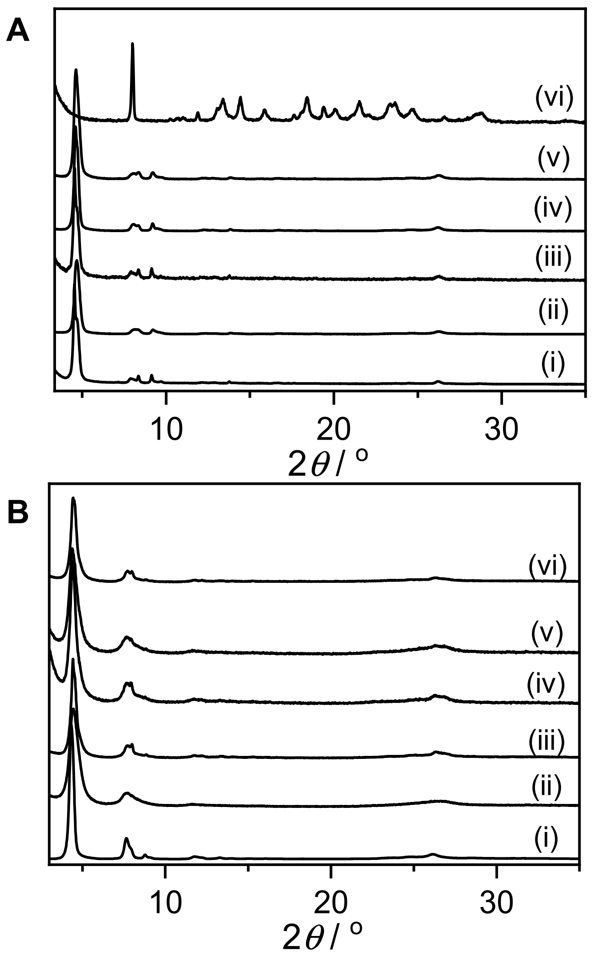

Supplementary Fig. 30. PXRD patterns of (A) **HOF-b** and (B) **MOF-b-Cu** of (i) as-synthesized, after the treatments under (ii) 3M HCl, (iii) 1M NaOH, (iv) boiling water, (v) 200 °C under N_2, (vi) 350 °C under vacuum (0.1 mmHg) for 2 days.

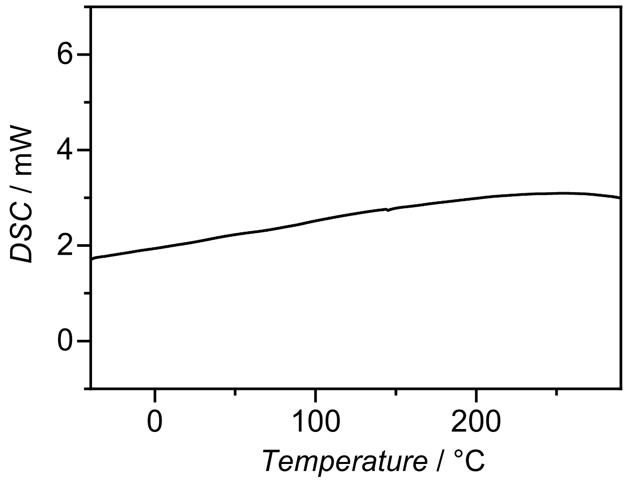

Supplementary Fig. 31. Differential scanning calorimetry of **MOF-b-Cu** from 228 K to 563 K.

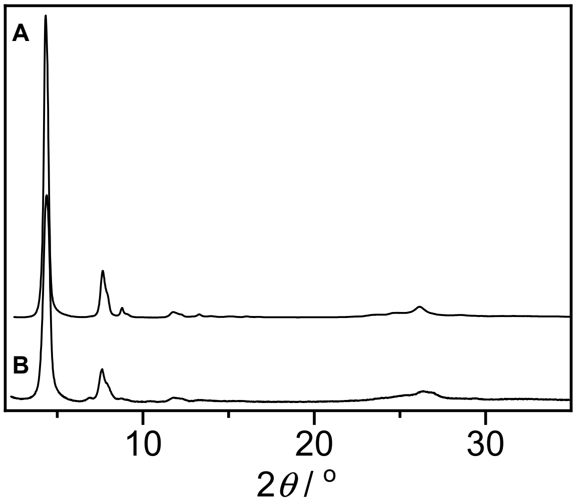

Supplementary Fig. 32. PXRD patterns of (A) **MOF-b-Cu** and (B) **MOF-b-Cu-338K**.

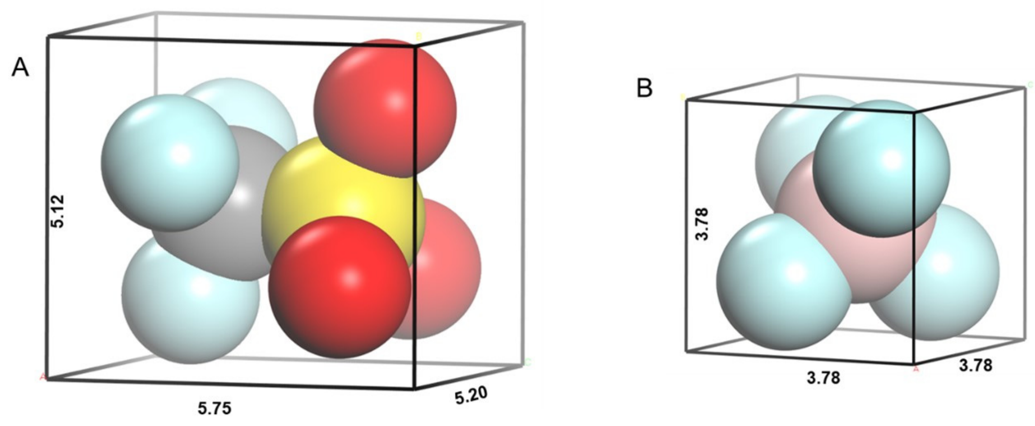

Supplementary Fig. 33. Molecular dimensions of (A) $CF_3SO_3^-$ and (B) BF_4^-. The unit of length is angstrom (Å). The molecular models were optimized using Materials Studio software through DMol3 module[11]. Atom colors: C, gray; S, yellow; F, light blue; O, red; B, pink.

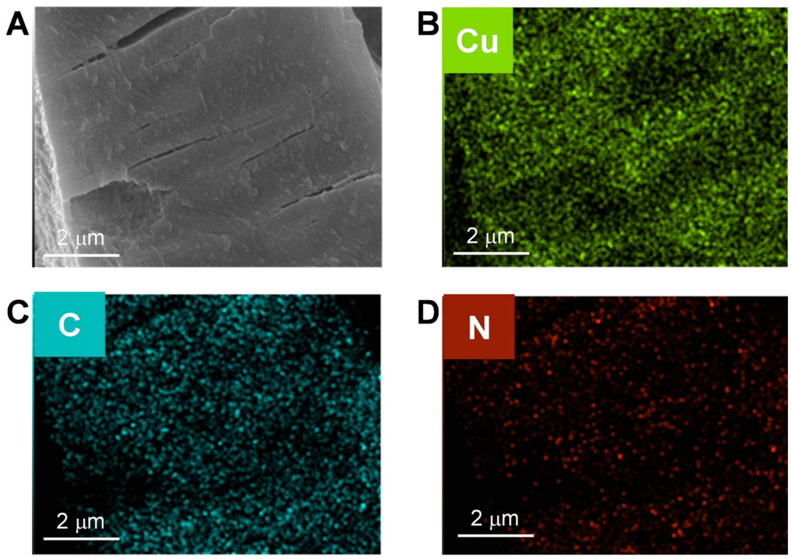

Supplementary Fig. 34. (A) SEM images and (B-D) EDS mapping images of **MOF-a-Cu**.

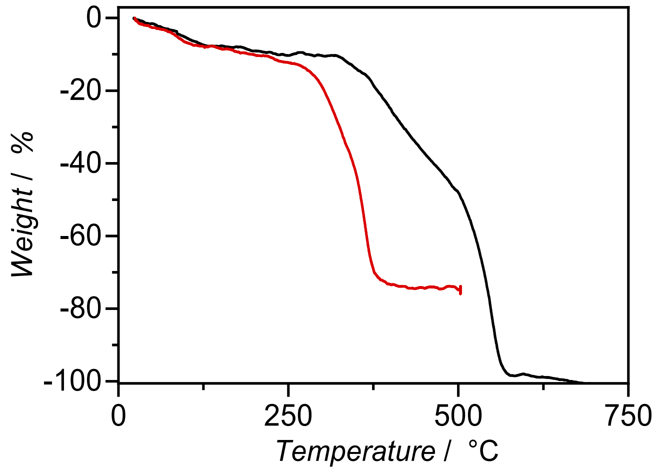

Supplementary Fig. 35. TGA profiles in air of (A) **HOF-a** (back curve) and (B) **MOF-a-Cu** (red curve).

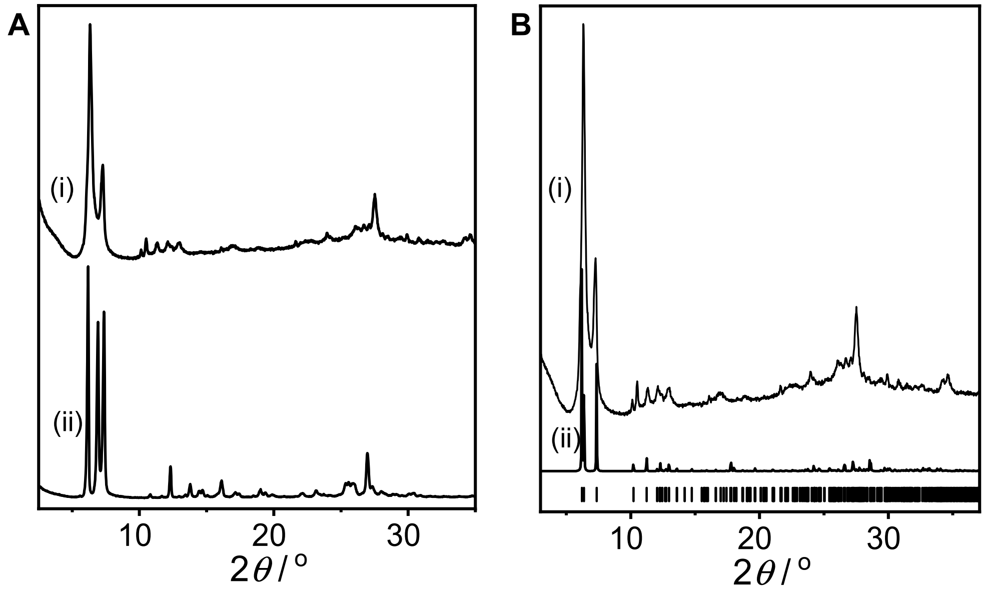

Supplementary Fig. 36. (A) PXRD patterns of (i) **MOF-a-Cu** and (ii) **HOF-a**. (B) (i) Experimental and (ii) simulated PXRD patterns of **MOF-a-Cu**.

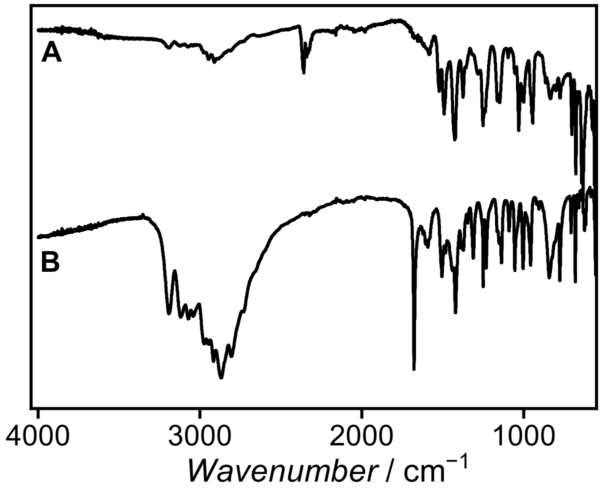

Supplementary Fig. 37. FT-IR spectra of (A) **MOF-a-Cu** and (B) **HOF-a**.

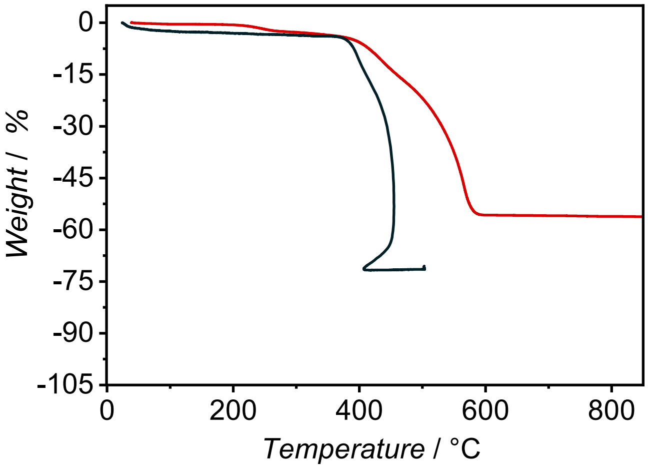

Supplementary Fig. 38. TGA profiles in air of (A) **MOF-b-Ag** (black curve) and (B) **MOF-b-Au** (red curve). The irregularity observed in the TGA profile of **MOF-b-Ag** at the temperature of 405 to 456 °C is due to its abrupt decomposition with an intense exothermic reaction.

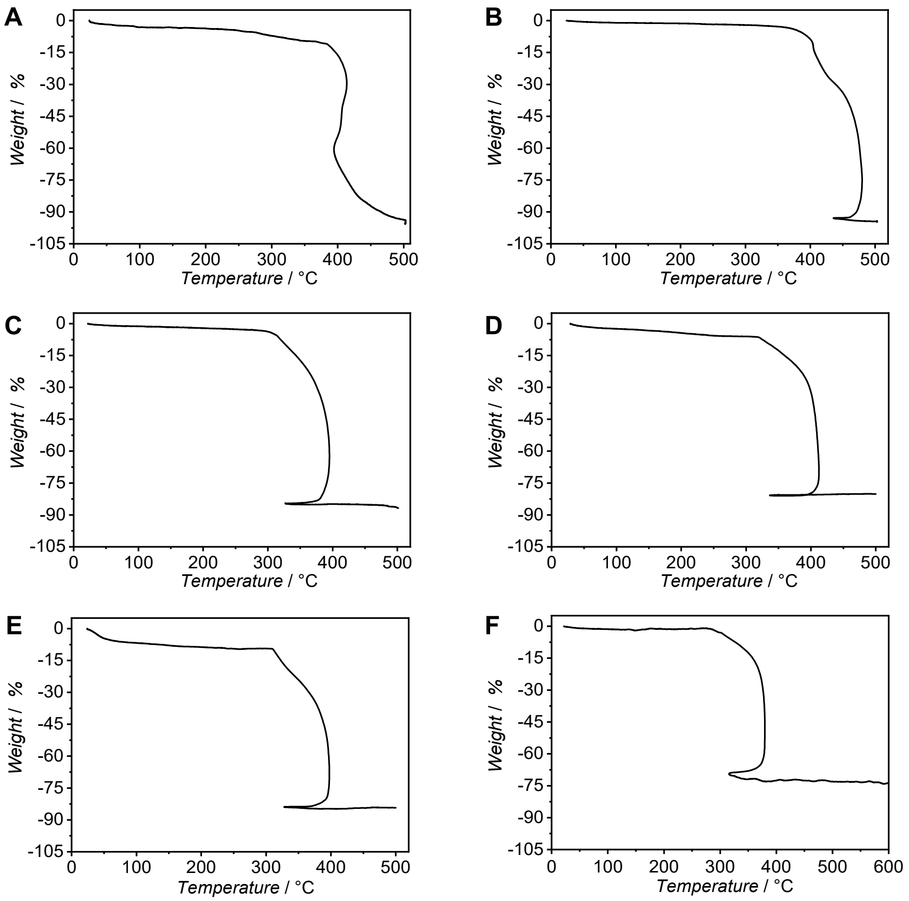

Supplementary Fig. 39. TGA profiles in air of **HOF|MOF-*x*h** where *x* is (A) 0.5, (B) 2.5, (C) 6, (D) 10, (E) 20, and (F) 48 (**MOF-b-Cu**). The irregularity observed in the TGA profiles of **HOF|MOF-*x*h** (*x* = 0.5, 2.5, 6, 10, 20, and 48) in air at the temperature of 300 to 400 °C is due to their abrupt decomposition with an intense exothermic reaction.

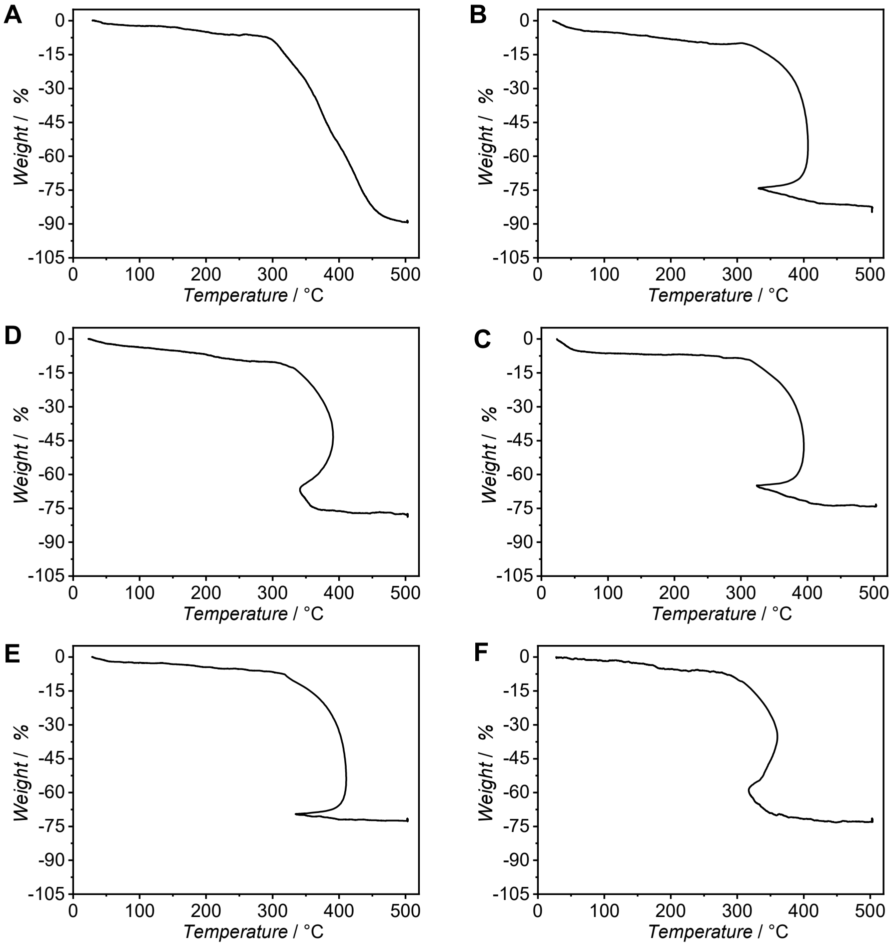

Supplementary Fig. 40. TGA profiles in air of **HOF|MOF-*x*h-BF_4** where *x* is (A) 0.5, (B) 2.5, (C) 6, (D) 10, (E) 14, and (F) 20. The irregularity observed in the TGA profiles of **HOF|MOF-*x*h-BF_4** (x = 2.5, 6, 10, 14, and 20) in air at the temperature of 300 to 400 °C is due to their abrupt decomposition with an intense exothermic reaction.

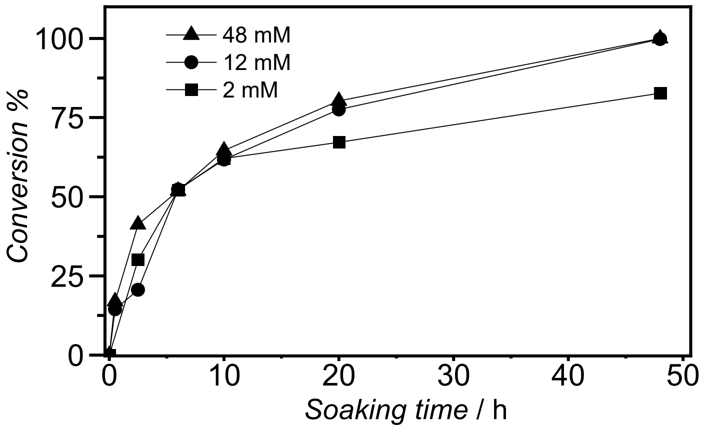

Supplementary Fig. 41. Metalation conversion during 48 h of 2 mM, 12 mM, and 48 mM $Cu(CF_3SO_3)$ solution.

Supplementary Table 3. The metalation conversion calculated by TGA profiles in air of **HOF|MOF-*x*h-BF₄** (x = 0.5, 2.5, 6, 10, 14, and 20).

Samples	Weight loss at 200 °C (wt%)	Weight loss at 500 °C (wt%)	Metal content (wt%)	Theoretical ($C_{13}H_{11}CuN_4$) (wt%)	Conv.
**HOF|MOF-0.5h-BF_4**	4.4%	89.4%	8.8%	22.1%	39.8%
**HOF|MOF-2.5h-BF_4**	7.7%	82.9%	14.8%	22.1%	67.0%
**HOF|MOF-6h-BF_4**	6.1%	77.5%	19.1%	22.1%	86.4%
**HOF|MOF-10h-BF_4**	7.0%	74.2%	22.1%	22.1%	~100%
**HOF|MOF-14h-BF_4**	4.2%	72.6%	22.8%	22.1%	~100%
**HOF|MOF-20h-BF_4**	5.3%	72.7%	23.0%	22.1%	~100%

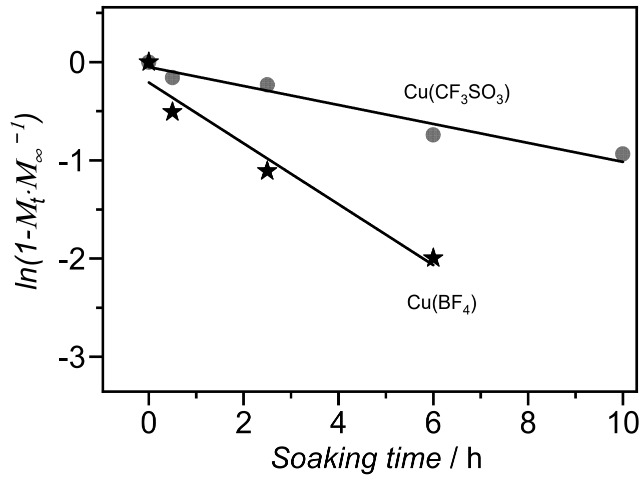

Supplementary Fig. 42. The kinetics profiles of $Cu(BF_4)$ and $Cu(CF_3SO_3)$ diffusion along the 1D channel at 298 K.

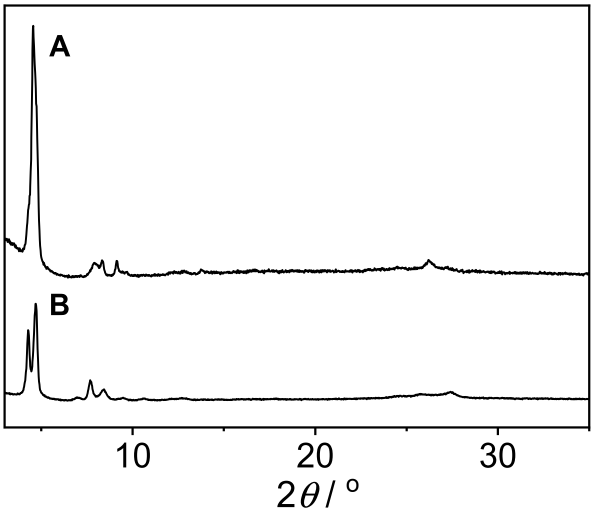

Supplementary Fig. 43. PXRD patterns of (A) **HOF|MOF-2.5h** and (B) **HOF|MOF-2.5h-BF_4**.

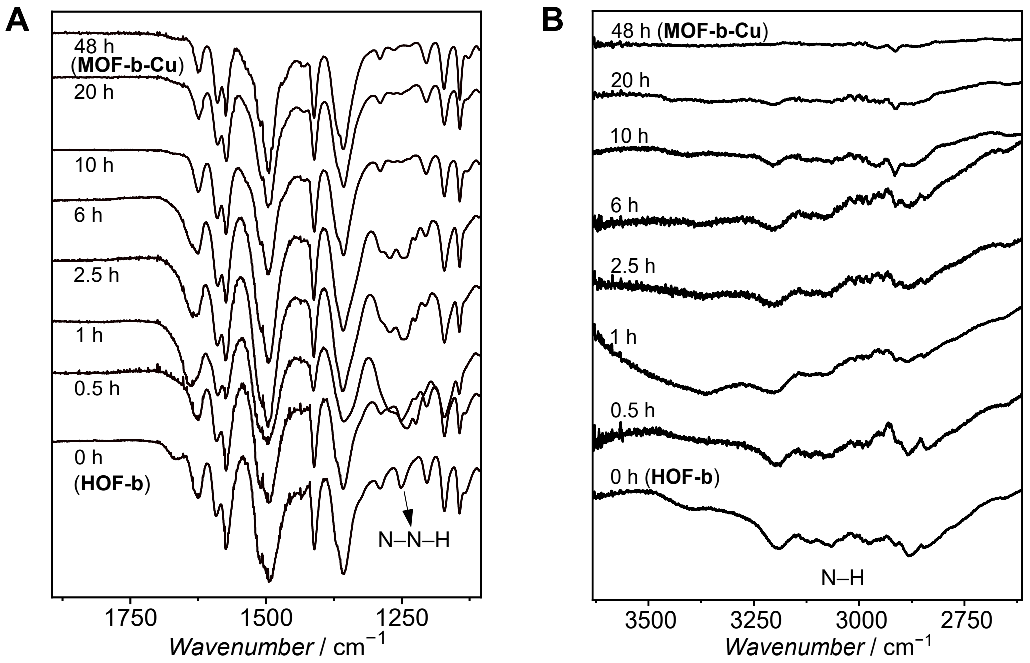

Supplementary Fig. 44. Time-course FT-IR spectra[12–14] of **HOF|MOF-*x*h** (x = 0, 0.5, 1, 2.5, 6, 10, 20, and 48) in the wavenumber of (A) 1850 to 1100 cm^{-1} and (B) 3600 to 2600 cm^{-1}.

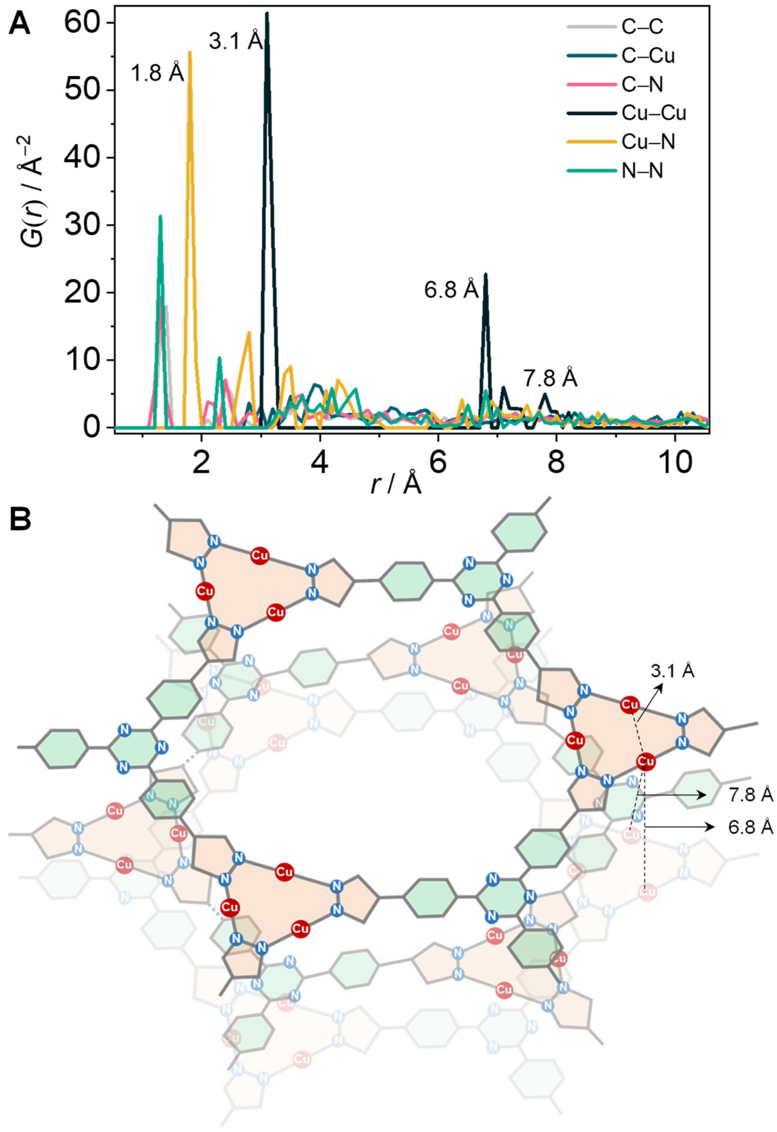

Supplementary Fig. 45. (A) Simulated PDF profiles with the peak assignment of **MOF-b-Cu**. (B) Schematics of layered structure of **MOF-b-Cu**.

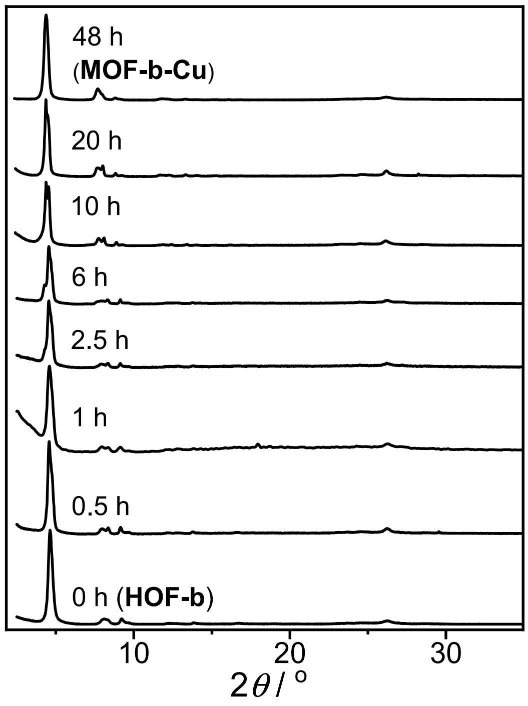

Supplementary Fig. 46. Time-course PXRD patterns of **HOF|MOF-*x*h** (x = 0, 0.5, 1, 2.5, 6, 10, 20, and 48).

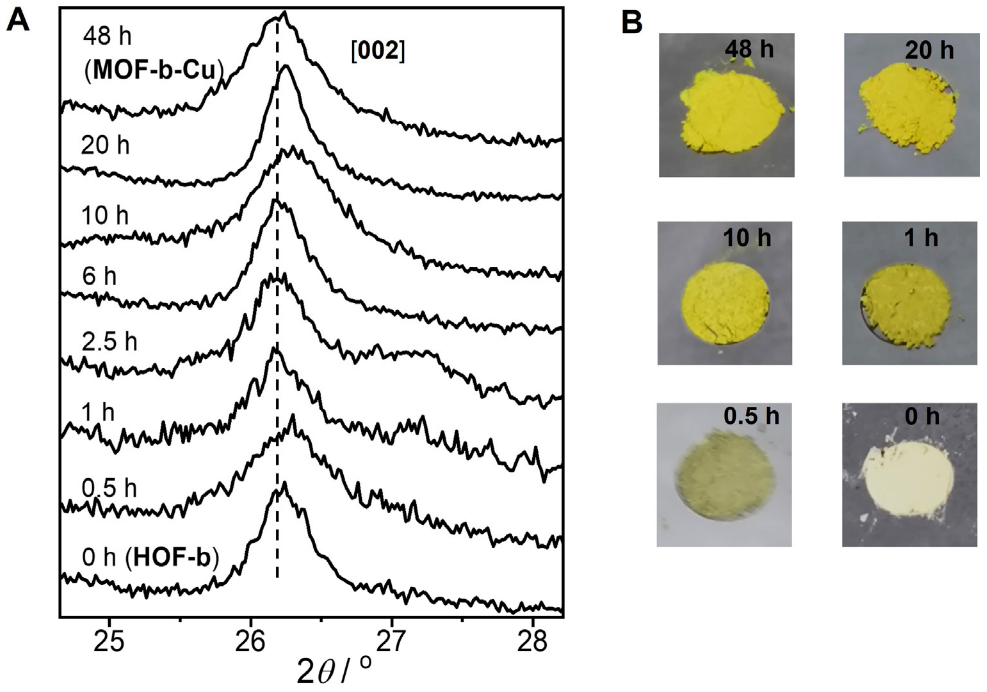

Supplementary Fig. 47. (A) PXRD patterns of **HOF|MOF-*x*h** (x = 0, 0.5, 1, 2.5, 6, 10, 20, and 48) in the two-theta of 25° to 28°. (B) Optical images of **HOF|MOF-*x*h** (x = 0, 0.5, 1, 10, 20, and 48) on the silicon support.

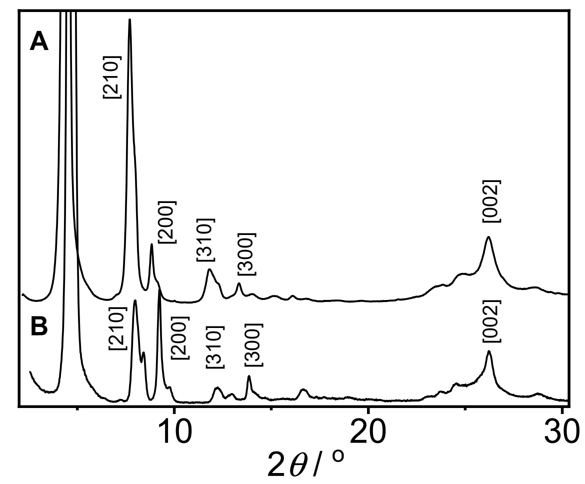

Supplementary Fig. 48. PXRD patterns of (A) **MOF-b-Cu** and (B) **HOF-b**.

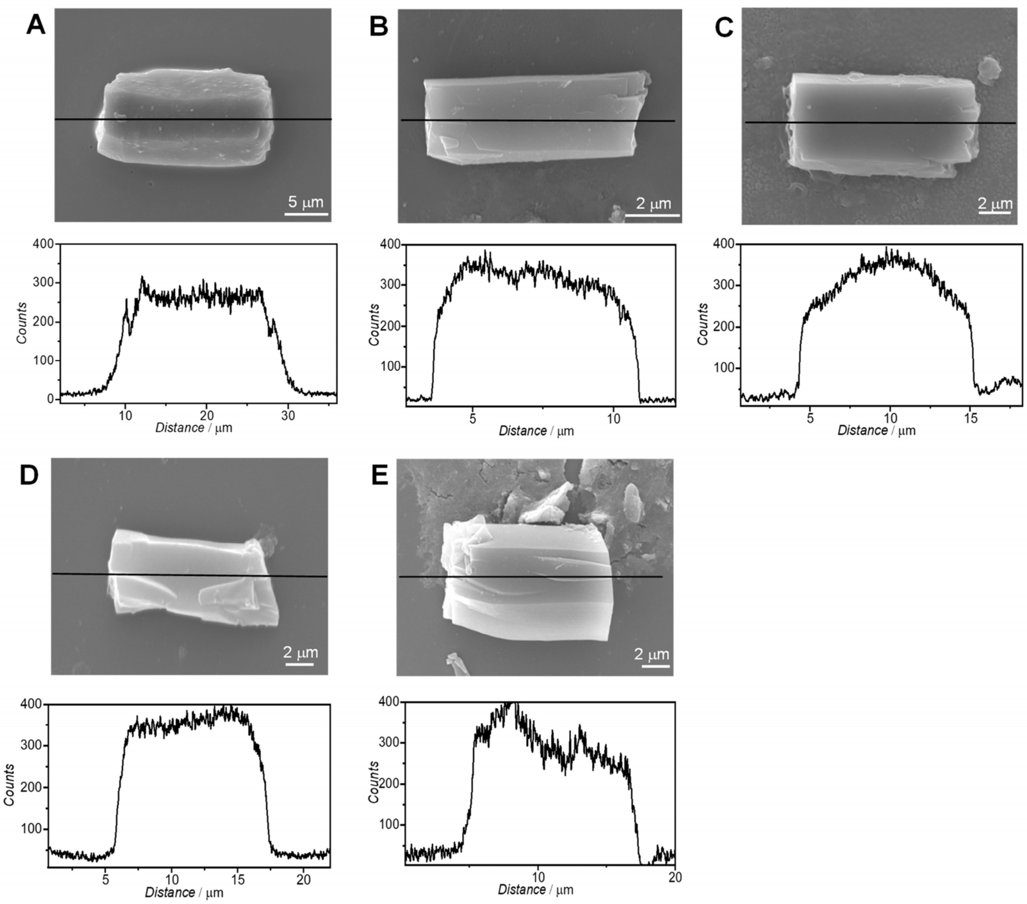

Supplementary Fig. 49. SEM images and corresponding EDS-line scan images for carbon element of (A) **HOF-b**, (B) **HOF|MOF-2.5h**, (C) **HOF|MOF-6h**, (D) **HOF|MOF-20h**, and (E) **MOF-b-Cu**.

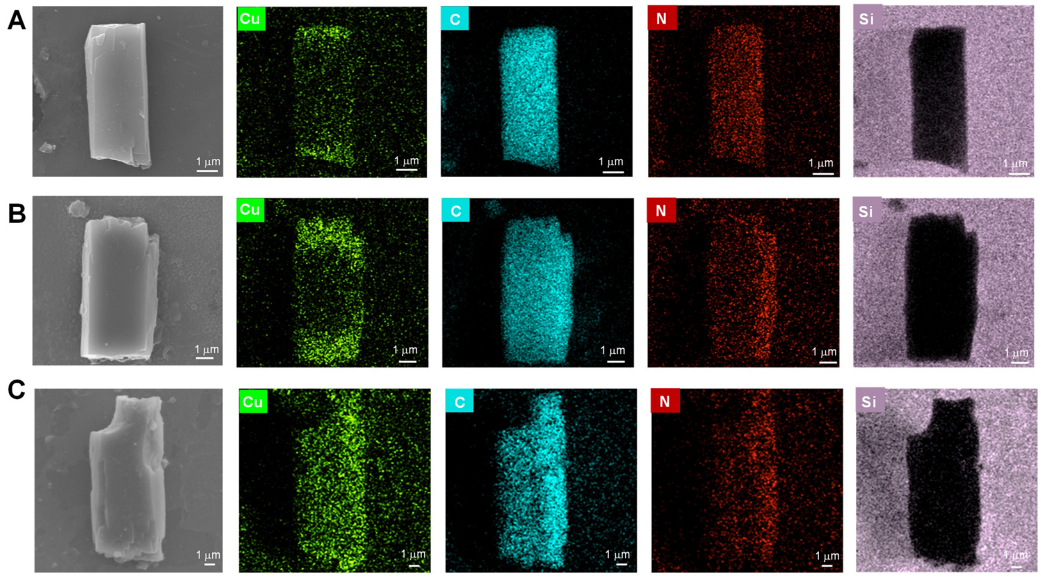

Supplementary Fig. 50. SEM images and corresponding EDS mapping images of (A) **HOF|MOF-2.5h**, (B) **HOF|MOF-6h**, and (C) **MOF-b-Cu**.

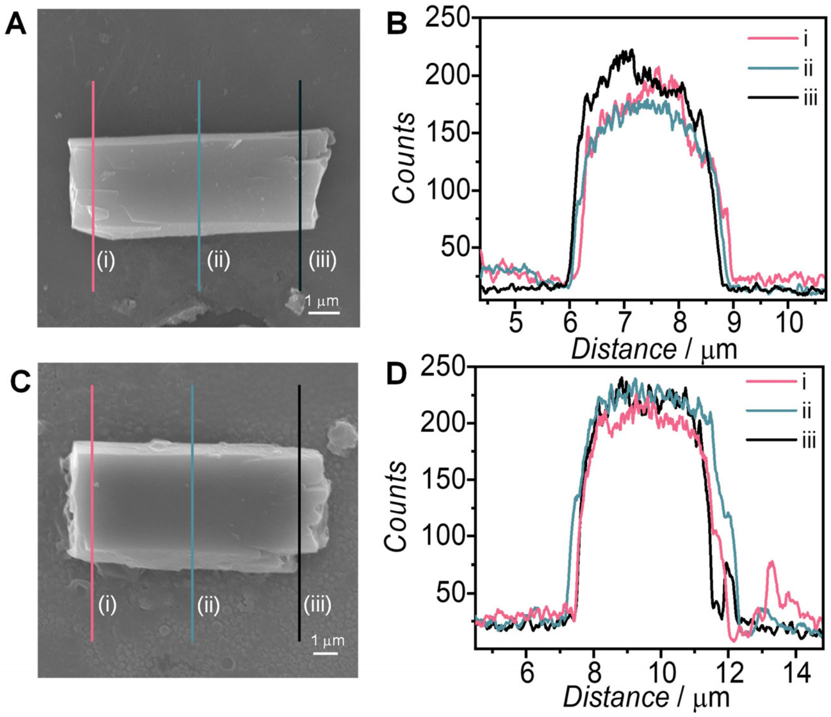

Supplementary Fig. 51. SEM images of (A) **HOF|MOF-2.5h** and (C) **HOF|MOF-6h**. Their corresponding EDS-line scan images for carbon element of (B) **HOF|MOF-2.5h** and (D) **HOF|MOF-6h** along the *ab* plane.

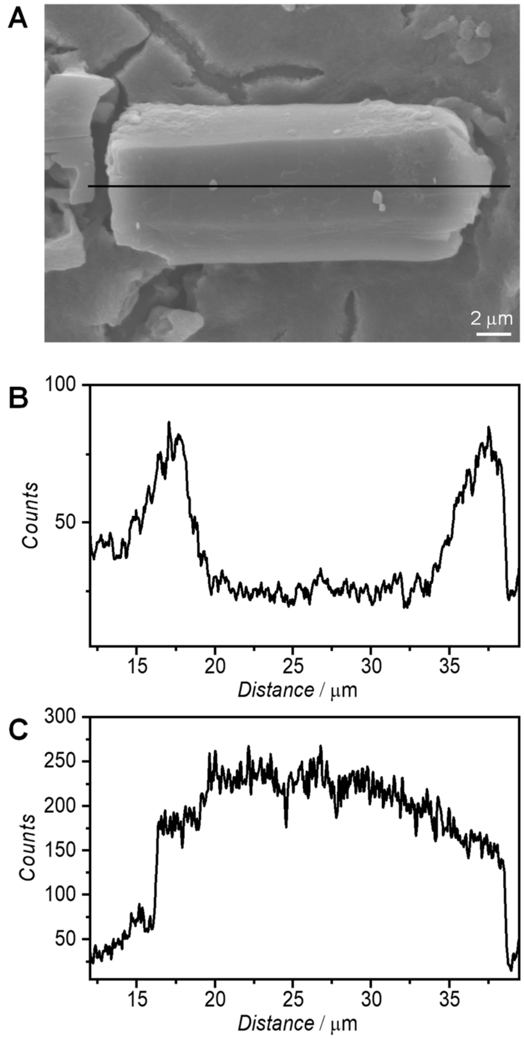

Supplementary Fig. 52. (A) SEM image of as-synthesized **HOF|MOF-6h**. Its corresponding SEM-EDS line scan images for (B) Cu element and (C) carbon element of as-synthesized **HOF|MOF-6h** (without solvent exchange and filtration).

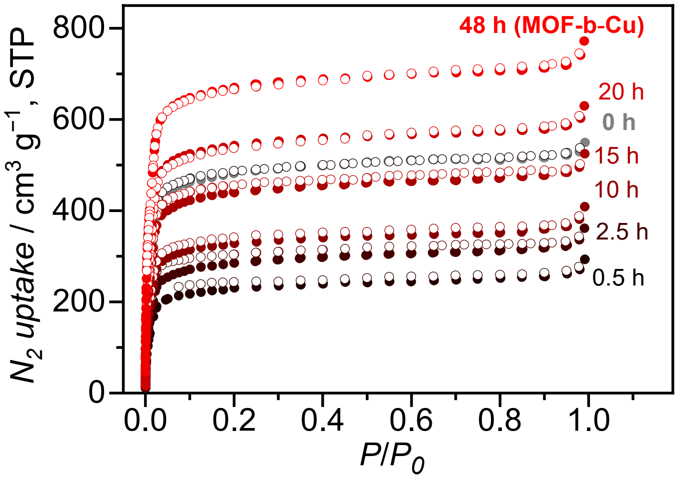

Supplementary Fig. 53. N_2 adsorption (●) and desorption (○) isotherms of **HOF|MOF-*x*h** (*x* = 0, 0.5, 2.5, 10, 15, 20, and 48) at 77 K.

Supplementary Table 4. The BET surface areas, NLDFT pore sizes by N_2 sorption isotherms of **HOF|MOF-*x*h** (*x* = 0, 0.5, 2.5, 6, 10, 20, and 48), **MOF-b-Ag**, and **MOF-b-Au**.

Samples	S_{BET} ($m^2\ g^{-1}$)	NLDFT pore size (nm)
**HOF-b** (0 h)	2105	1.61
**HOF|MOF-0.5h**	893	1.91
**HOF|MOF-2.5h**	1270	1.87
**HOF|MOF-6h**	1410	1.87
**HOF|MOF-10h**	1600	1.87
**HOF|MOF-15h**	1940	1.87
**HOF|MOF-20h**	2388	1.87
**MOF-b-Cu** (48 h)	2900	1.87
**MOF-b-Ag**	1954	1.87
**MOF-b-Au**	1379	1.87

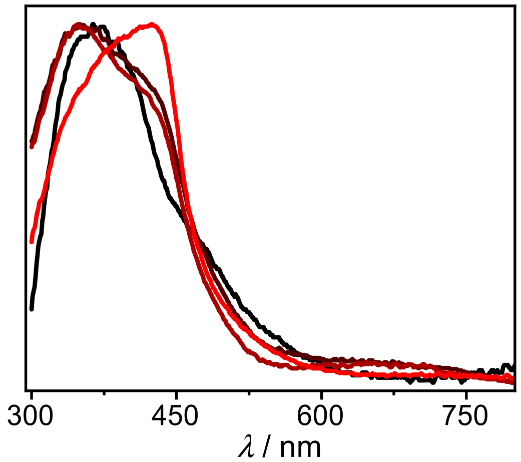

Supplementary Fig. 54. Normalized solid-state UV-vis spectra of (A) **HOF-b** (black curve), (B) **HOF|MOF-2.5h** (dark red curve), (C) **HOF|MOF-6h** (deep red curve), and (D) **MOF-b-Cu** (red curve).

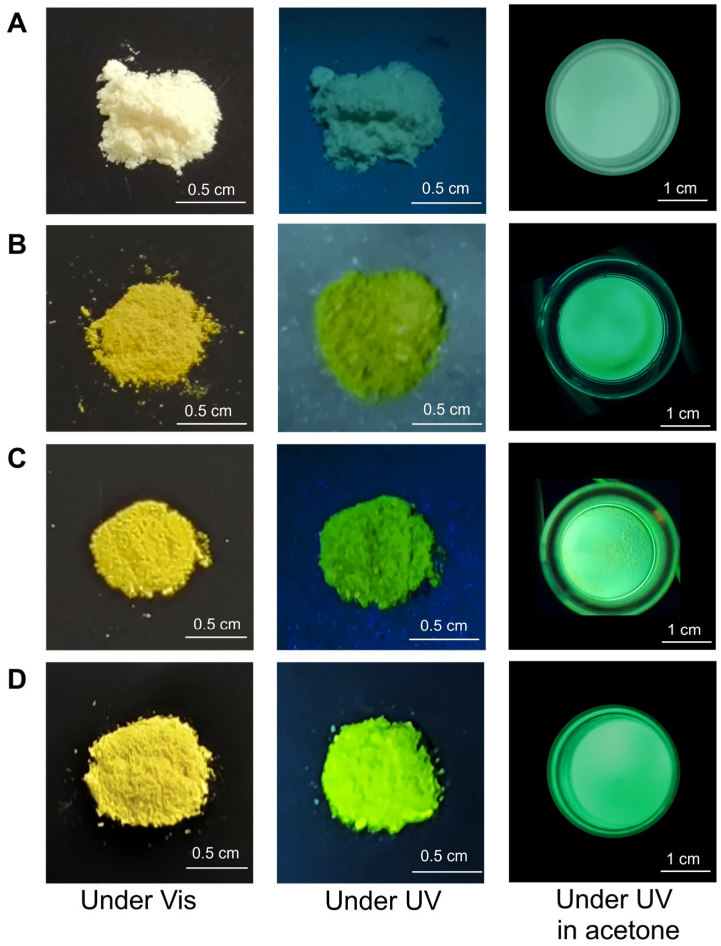

Supplementary Fig. 55. Optical images of (A) **HOF-b**, (B) **HOF|MOF-2.5h**, (C) **HOF|MOF-6h**, and (D) **MOF-b-Cu** upon visible light (Vis) and UV excitation in the air at 365 nm.

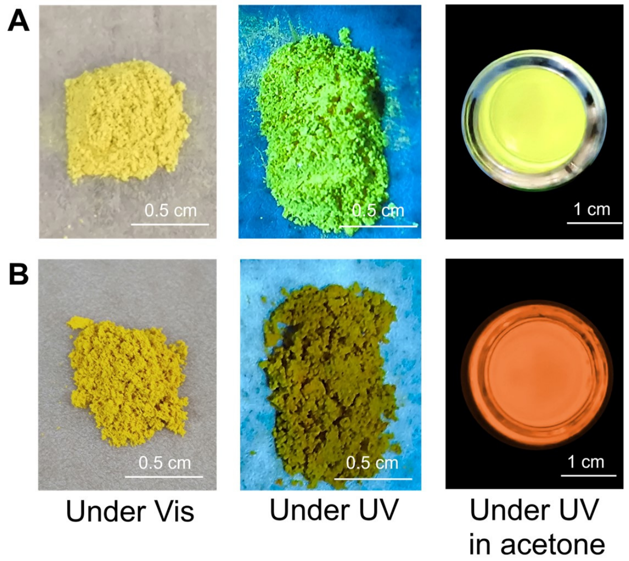

Supplementary Fig. 56. Optical images of (A) **MOF-b-Ag** and (B) **MOF-b-Au** upon Vis and UV excitation in the air at 365 nm.

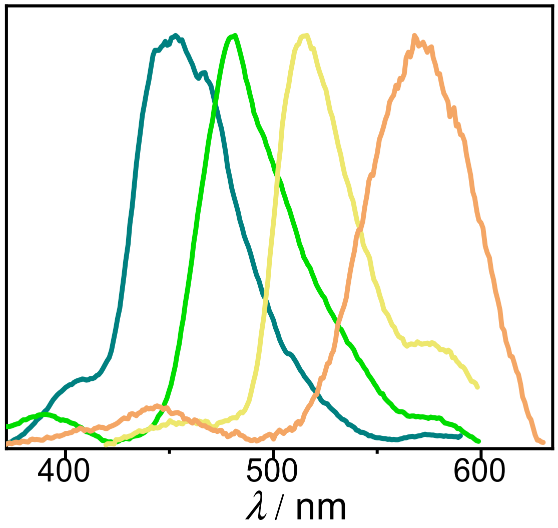

Supplementary Fig. 57. Normalized steady-state photoluminescence spectra of samples in acetone of (A) **HOF-b** (cyan curve, $\lambda_{ex} = 300$ nm), (B) **MOF-b-Cu** (green curve, $\lambda_{ex} = 300$ nm), (C) **MOF-b-Ag** (yellow curve, $\lambda_{ex} = 300$ nm), and (D) **MOF-b-Au** (orange curve, $\lambda_{ex} = 340$ nm).

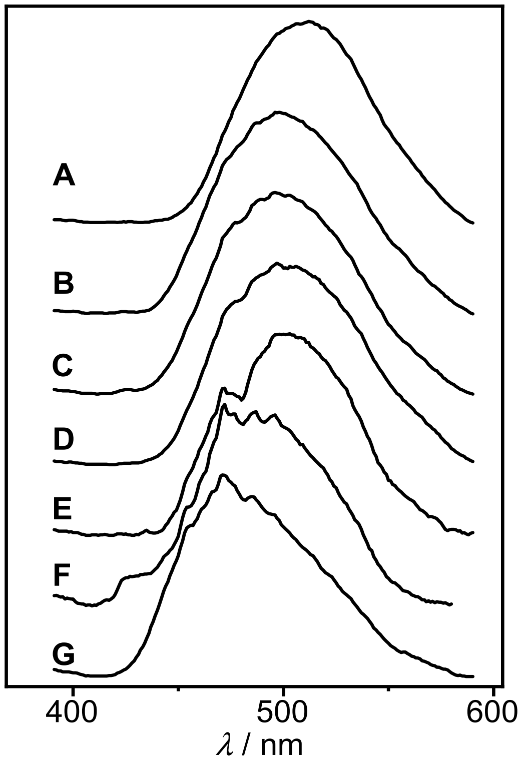

Supplementary Fig. 58. Time-course normalized steady-state photoluminescence spectra (λ_{ex} = 300 nm) of **HOF|MOF-*x*h** where *x* is (A) 48 (**MOF-b-Cu**), (B) 20, (C) 10, (D) 6, (E) 2.5, (F) 1, and (G) 0 (**HOF-b**).

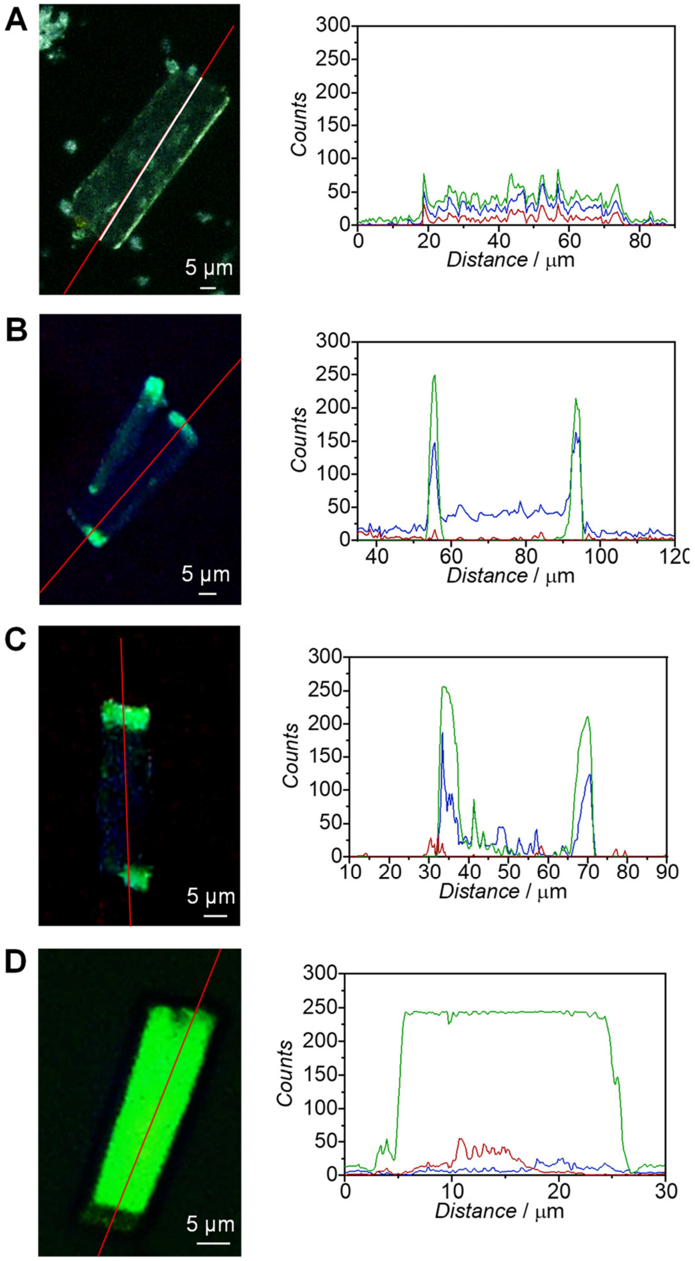

Supplementary Fig. 59. Emission images of (A) **HOF-b**, (B) **HOF|MOF-2.5h**, (C) **HOF|MOF-6h**, and (D) **MOF-b-Cu** upon UV excitation in air at 365 nm and corresponding color component of the emission can be resolved by RGB (Red, red curve; Green, green curve; Blue, blue curve) acquisition.

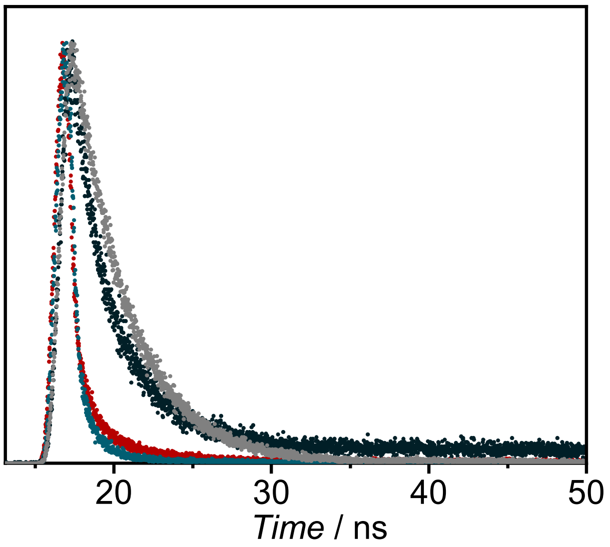

Supplementary Fig. 60. Photoluminescence lifetime measurements of (A) **HOF-b** (cyan dots), (B) **MOF-b-Cu** (red dots), (C) **MOF-b-Ag** (black dots), and (D) **MOF-b-Au** (gray dots) in air.

Supplementary Table 5. Photoluminescence property.

Samples	λ_{ex}	Emission peaks[a]	Emission peaks[b]	QY%	Average lifetime
**HOF-b**	300 nm	465 nm	446 nm	2.8	0.83 ns
**HOF|MOF-2.5h**	300 nm	470 nm, 502 nm	469 nm	1.7	
**HOF|MOF-6h**	300 nm	475 nm, 504 nm	474 nm	3.3	
**MOF-b-Cu**	300 nm	505 nm	475 nm	4.9	1.14 ns
**MOF-b-Ag**	300 nm	519 nm	514 nm	5.8	4.04 ns
**MOF-b-Au**	340 nm	545 nm	578 nm	3.7	3.28 ns

[a]Guest-free samples. [b]Samples in acetone.

Supplementary Table 6. Solvent polarity and photoluminescence property (λ_{ex} = 300 nm).

**MOF-b-Cu** @solvent	Solvent	$E_T(30)$ (kcal mol^{-1})[15, 16]	Emission peaks (nm)
aprotic	n-hexane	30.9	456
	1,4-dioxane	36.0	463
	tetrahydrofuran	37.5	468
	chloroform	39.1	470
	acetone	42.2	475
protic	2-propanol	48.4	469
	ethanol	51.9	478
	methanol	55.4	490
	2,2,2-trifluoroethanol	59.8	496
	H_2O	63.1	502

Supplementary Table 7. SCXRD data and structure refinement for **HOF-a**.

ORTEP plot	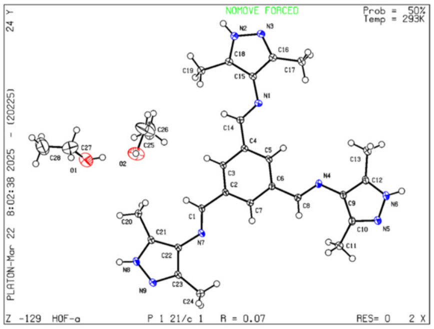
CCDC number	2433227
Empirical formula	$C_{28}H_{38}N_9O_2$
Formula weight	532.67
Temperature/K	293(2)
Crystal system	monoclinic
Space group	$P2_1$/c (No.14)
a/Å	12.7163(6)
b/Å	29.2334(11)
c/Å	7.6434(3)
α/°	90
β/°	90.169(4)
γ/°	90
Volume/Å^3	2841.3(2)
Z	4
ρ_{calc}/g cm^{-3}	1.245
μ/mm^{-1}	0.083
F(000)	1140
Crystal size/mm^3	0.08 × 0.04 × 0.02
Radiation	Mo Kα (λ = 0.71073)
2θ range for data collection/°	5.266 to 63.668
Index ranges	-14 ≤ h ≤ 18, -42 ≤ k ≤ 42, -11 ≤ l ≤ 11
Reflections collected	20681
Independent reflections	7930 [R_{int} = 0.0290, R_{sigma} = 0.0488]
Data/restraints/parameters	7930/0/362
Goodness-of-fit on F^2	1.031
Final R indexes [I>=2σ (I)]	R_1 = 0.0681, wR_2 = 0.1626
Final R indexes [all data]	R_1 = 0.1009, wR_2 = 0.1801
Largest diff. peak/hole / e Å^{-3}	0.54/-0.36

Supplementary Table 8. Fractional atomic coordinates and the unit cell of **HOF-b**.

Chemical Formula		$C_{13}H_{12}N_4$		
Z		6		
Space Group		$P6_3$ (No.173)		
Crystal System		Hexagonal		
a = *b*		21.9873 Å		
c		6.9167 Å		
Cell Volume		2895.832 Å^3		
Asymmetric Unit		29 sites		
Unit Cell		174 sites		
ρ_{calc}		0.7716 g cm^{-3}		
**Sites**	**Occupy**	***x***	***y***	***z***
C4	C1.00	0.5356	0.4734	0.0264
C5	C1.00	0.8729	0.436	0.0231
C6	C1.00	0.823	0.3645	-0.0044
C7	C1.00	0.8346	0.4697	0.045
C8	C1.00	0.0463	0.5578	0.049
C9	C1.00	0.0925	0.6308	0.0529
C10	C1.00	0.1642	0.6589	0.0557
C11	C1.00	0.1934	0.6137	0.054
C12	C1.00	0.1467	0.5397	0.0505
C13	C1.00	0.0753	0.5134	0.0481
C14	C1.00	0.2655	0.6411	0.0539
C16	C1.00	0.4561	0.3104	0.0808
C17	C1.00	0.6959	0.5307	-0.0353
H18	H1.00	0.5723	0.5301	0.0116
H19	H1.00	0.0725	0.6676	0.0506
H20	H1.00	0.1987	0.7162	0.0579
H21	H1.00	0.1675	0.5034	0.0487
H22	H1.00	0.0405	0.4562	0.0445
H23	H1.00	0.4502	0.2618	0.1437
H24	H1.00	0.4248	0.296	-0.0526
H25	H1.00	0.433	0.3305	0.1852
H26	H1.00	0.7406	0.5313	-0.1087
H27	H1.00	0.7151	0.5585	0.1033
H28	H1.00	0.6794	0.5609	-0.1261
H29	H1.00	0.5771	0.3	0.0336

N1	N1.00	0.6454	0.403	0
N2	N1.00	0.5817	0.3477	0.0294
N3	N1.00	0.4728	0.4456	0.0433
N15	N1.00	0.3063	0.7112	0.0534

Supplementary Table 9. Fractional atomic coordinates and the unit cell of **MOF-b-Cu**.

Chemical Formula	$C_{13}H_{11}CuN_4$			
Z	6			
Space Group	*P*3 (No.143)			
Crystal System	Trigonal			
a = *b*	23.8643 Å			
c	6.9033 Å			
Cell Volume	3404.746 Å^3			
Asymmetric Unit	58 sites			
Unit Cell	174 sites			
ρ_{calc}	0.8393 g cm^{-3}			
**Sites**	**Occupy**	***x***	***y***	***z***
C4	C1.00	0.5479	0.501	0.0334
C5	C1.00	0.8825	0.4236	0.0334
C6	C1.00	0.8371	0.359	0.0334
C7	C1.00	0.846	0.4535	0.0334
C8	C1.00	0.0604	0.5439	0.0334
C9	C1.00	0.0979	0.6114	0.0334
C10	C1.00	0.1654	0.6417	0.0334
C11	C1.00	0.1963	0.6049	0.0334
C12	C1.00	0.1588	0.5372	0.0334
C13	C1.00	0.0913	0.5068	0.0334
C14	C1.00	0.2675	0.637	0.0334
C16	C1.00	0.4755	0.3464	0.0334
C17	C1.00	0.6954	0.5462	0.0334
C21	C1.00	0.4521	0.499	0.5334
C22	C1.00	0.1176	0.5765	0.5334
C23	C1.00	0.1629	0.641	0.5334
C24	C1.00	0.1541	0.5465	0.5334
C25	C1.00	0.9396	0.4562	0.5334
C26	C1.00	0.9021	0.3887	0.5334
C27	C1.00	0.8346	0.3583	0.5334

C28	C1.00	0.8037	0.3951	0.5334
C29	C1.00	0.8413	0.4628	0.5334
C30	C1.00	0.9087	0.4932	0.5334
C31	C1.00	0.7325	0.363	0.5334
C33	C1.00	0.5245	0.6536	0.5334
C34	C1.00	0.3046	0.4538	0.5334
Cu35	Cu1.00	0.2982	0.7158	0.5334
Cu36	Cu1.00	0.7018	0.2842	0.0334
H37	H1.00	0.5816	0.5556	0.0334
H38	H1.00	0.0734	0.6419	0.0334
H39	H1.00	0.1957	0.6968	0.0334
H40	H1.00	0.1833	0.5067	0.0334
H41	H1.00	0.0612	0.4518	0.0334
H42	H1.00	0.4654	0.298	0.0922
H43	H1.00	0.4562	0.3399	-0.1196
H44	H1.00	0.4504	0.3661	0.1277
H45	H1.00	0.7143	0.5611	-0.1175
H46	H1.00	0.7355	0.5506	0.1286
H47	H1.00	0.6782	0.5791	0.0892
H48	H1.00	0.4184	0.4444	0.5334
H49	H1.00	0.9266	0.3581	0.5334
H50	H1.00	0.8044	0.3032	0.5334
H51	H1.00	0.8168	0.4933	0.5334
H52	H1.00	0.9388	0.5483	0.5334
H53	H1.00	0.5415	0.6706	0.3804
H54	H1.00	0.5507	0.6287	0.5922
H55	H1.00	0.5358	0.6968	0.6277
H56	H1.00	0.3168	0.4234	0.6286
H57	H1.00	0.2956	0.4339	0.3825
H58	H1.00	0.2596	0.4519	0.5892
N1	N1.00	0.6492	0.4262	0.0334
N2	N1.00	0.591	0.3734	0.0334
N3	N1.00	0.4858	0.4772	0.0334
N15	N1.00	0.3038	0.7026	0.0334
N18	N1.00	0.3508	0.5738	0.5334
N19	N1.00	0.409	0.6266	0.5334
N20	N1.00	0.5142	0.5228	0.5334

N32	N1.00	0.6962	0.2974	0.5334

Supplementary Table 10. Fractional atomic coordinates and the unit cell of **MOF-b-Ag**.

Chemical Formula	$C_{13}H_{11}AgN_4$			
Z	6			
Space Group	*P*3 (No.143)			
Crystal System	Trigonal			
a = *b*	24.6921 Å			
c	6.8753 Å			
Cell Volume	3630.265 Å^3			
Asymmetric Unit	58 sites			
Unit Cell	174 sites			
ρ_{calc}	0.9089 g cm^{-3}			
**Sites**	**Occupy**	***x***	***y***	***z***
Ag35	Ag1.00	0.296	0.7201	0.5334
Ag36	Ag1.00	0.7024	0.2795	0.0334
C4	C1.00	0.5499	0.5121	0.0334
C5	C1.00	0.8944	0.4259	0.0334
C6	C1.00	0.8483	0.3632	0.0334
C7	C1.00	0.8625	0.4588	0.0334
C8	C1.00	0.0685	0.5398	0.0334
C9	C1.00	0.1028	0.6052	0.0334
C10	C1.00	0.1682	0.6368	0.0334
C11	C1.00	0.2005	0.6033	0.0334
C12	C1.00	0.1659	0.5377	0.0334
C13	C1.00	0.1008	0.5062	0.0334
C14	C1.00	0.2696	0.6363	0.0334
C16	C1.00	0.4719	0.3608	0.0334
C17	C1.00	0.6918	0.5499	0.0334
C21	C1.00	0.4487	0.4873	0.5334
C22	C1.00	0.1067	0.5763	0.5334
C23	C1.00	0.1524	0.639	0.5334
C24	C1.00	0.14	0.5445	0.5334
C25	C1.00	0.9322	0.4613	0.5334
C26	C1.00	0.8979	0.3958	0.5334
C27	C1.00	0.8324	0.364	0.5334
C28	C1.00	0.7999	0.3972	0.5334

C29	C1.00	0.8344	0.4629	0.5334
C30	C1.00	0.8997	0.4946	0.5334
C31	C1.00	0.7305	0.3639	0.5334
C33	C1.00	0.5247	0.6389	0.5334
C34	C1.00	0.3061	0.4487	0.5334
H37	H1.00	0.5835	0.5615	0.0334
H38	H1.00	0.0788	0.6317	0.0334
H39	H1.00	0.1933	0.6874	0.0334
H40	H1.00	0.1892	0.5104	0.0334
H41	H1.00	0.0767	0.4558	0.0334
H42	H1.00	0.461	0.3114	0.0334
H43	H1.00	0.451	0.3692	-0.0977
H44	H1.00	0.451	0.3692	0.1645
H45	H1.00	0.6905	0.5752	-0.0984
H46	H1.00	0.736	0.5484	0.0334
H47	H1.00	0.6905	0.5752	0.1652
H48	H1.00	0.4153	0.4379	0.5334
H49	H1.00	0.922	0.3694	0.5334
H50	H1.00	0.8075	0.3134	0.5334
H51	H1.00	0.811	0.4901	0.5334
H52	H1.00	0.9236	0.545	0.5334
H53	H1.00	0.5458	0.6307	0.4025
H54	H1.00	0.5458	0.6307	0.6643
H55	H1.00	0.5353	0.6882	0.5334
H56	H1.00	0.3074	0.4234	0.6652
H57	H1.00	0.3074	0.4234	0.4016
H58	H1.00	0.2619	0.4502	0.5334
N1	N1.00	0.6419	0.4333	0.0334
N2	N1.00	0.5856	0.3832	0.0334
N3	N1.00	0.4902	0.4915	0.0334
N15	N1.00	0.3032	0.6998	0.0334
N18	N1.00	0.3564	0.5654	0.5334
N19	N1.00	0.4132	0.6148	0.5334
N20	N1.00	0.5086	0.5081	0.5334
N32	N1.00	0.697	0.3004	0.5334

Supplementary Table 11. Fractional atomic coordinates and the unit cell of **MOF-b-Au**.

Chemical Formula	$C_{13}H_{11}AuN_4$			
Z	6			
Space Group	*P*3 (No.143)			
Crystal System	Trigonal			
a = *b*	24.4664 Å			
c	6.8698 Å			
Cell Volume	3561.352 $Å^3$			
Asymmetric Unit	58 sites			
Unit Cell	174 sites			
ρ_{calc}	1.1757 g cm^{-3}			
**Sites**	**Occupy**	***x***	***y***	***z***
Au35	Au1.00	0.2944	0.7173	0.5334
Au36	Au1.00	0.704	0.2823	0.0334
C4	C1.00	0.5512	0.51	0.0334
C5	C1.00	0.8904	0.4243	0.0334
C6	C1.00	0.8439	0.3608	0.0334
C7	C1.00	0.8581	0.4578	0.0334
C8	C1.00	0.066	0.5392	0.0334
C9	C1.00	0.1009	0.6052	0.0334
C10	C1.00	0.1668	0.637	0.0334
C11	C1.00	0.1993	0.6031	0.0334
C12	C1.00	0.1643	0.5369	0.0334
C13	C1.00	0.0985	0.5052	0.0334
C14	C1.00	0.2691	0.6362	0.0334
C16	C1.00	0.4724	0.357	0.0334
C17	C1.00	0.6946	0.5485	0.0334
C21	C1.00	0.4474	0.4895	0.5334
C22	C1.00	0.1108	0.5779	0.5334
C23	C1.00	0.1569	0.6414	0.5334
C24	C1.00	0.1445	0.5457	0.5334
C25	C1.00	0.9347	0.4619	0.5334
C26	C1.00	0.8999	0.3958	0.5334
C27	C1.00	0.8338	0.3638	0.5334
C28	C1.00	0.8011	0.3975	0.5334
C29	C1.00	0.836	0.4637	0.5334
C30	C1.00	0.9019	0.4956	0.5334

C31	C1.00	0.7311	0.364	0.5334
C33	C1.00	0.5241	0.6427	0.5334
C34	C1.00	0.3032	0.4501	0.5334
H37	H1.00	0.5859	0.5629	0.0334
H38	H1.00	0.0755	0.6336	0.0334
H39	H1.00	0.1947	0.6908	0.0334
H40	H1.00	0.1897	0.5085	0.0334
H41	H1.00	0.071	0.4514	0.0334
H42	H1.00	0.4614	0.3074	0.0746
H43	H1.00	0.4529	0.3562	-0.1179
H44	H1.00	0.4497	0.3744	0.1435
H45	H1.00	0.7043	0.5678	-0.1215
H46	H1.00	0.7378	0.5477	0.0916
H47	H1.00	0.6841	0.58	0.1301
H48	H1.00	0.4128	0.4365	0.5334
H49	H1.00	0.9253	0.3675	0.5334
H50	H1.00	0.8061	0.31	0.5334
H51	H1.00	0.8106	0.4921	0.5334
H52	H1.00	0.9292	0.5494	0.5334
H53	H1.00	0.54	0.664	0.3821
H54	H1.00	0.5491	0.6156	0.5747
H55	H1.00	0.5365	0.6821	0.6434
H56	H1.00	0.3182	0.4159	0.5916
H57	H1.00	0.2847	0.4361	0.3785
H58	H1.00	0.2645	0.4483	0.6301
N1	N1.00	0.6441	0.4308	0.0334
N2	N1.00	0.5874	0.3803	0.0334
N3	N1.00	0.4909	0.4892	0.0334
N15	N1.00	0.3031	0.7002	0.0334
N18	N1.00	0.3542	0.568	0.5334
N19	N1.00	0.4114	0.6179	0.5334
N20	N1.00	0.5078	0.5104	0.5334
N32	N1.00	0.6971	0.2999	0.5334

Supplementary Table 12. Fractional atomic coordinates and the unit cell of **MOF-a-Cu**.

Chemical Formula		$C_8H_8CuN_3$		
Z		6		
Space Group		*P*1		
Crystal System		Triclinic		
a		17.4550 Å		
b		17.3501 Å		
c		7.9473 Å		
α		86.662°		
β		59.284°		
γ		118.946°		
Cell Volume		1654.691 Å^3		
Asymmetric Unit		120 sites		
Unit Cell		120 sites		
ρ_{calc}		1.2628 g cm^{-3}		
**Sites**	**Occupy**	***x***	***y***	***z***
C3	C1.00	0.5492	0.1458	0.058
C5	C1.00	0.4702	0.5268	-0.0268
C6	C1.00	0.3717	0.4863	-0.0003
C7	C1.00	0.5171	0.6237	-0.0825
C8	C1.00	0.55	0.3652	0.0317
C9	C1.00	0.6402	0.4264	0.0303
C10	C1.00	0.2246	-0.0727	0.2411
C11	C1.00	0.604	0.0003	-0.1161
C22	C1.00	0.7945	0.4628	0.0364
C24	C1.00	0.4244	-0.0093	0.0772
C25	C1.00	0.4836	-0.0465	-0.0085
C26	C1.00	0.319	-0.0795	0.1449
C27	C1.00	0.5778	0.2413	0.0543
C28	C1.00	0.5191	0.2728	0.0441
C29	C1.00	0.9905	0.3746	0.0316
C30	C1.00	0.9992	0.6712	-0.0715
C41	C1.00	0.4891	0.3975	0.0146
C43	C1.00	0.9579	0.502	0.0021
C44	C1.00	1.0171	0.5984	-0.0304
C45	C1.00	1.0165	0.4718	0.0094
C46	C1.00	0.6988	0.3957	0.0426

C47	C1.00	0.6671	0.3029	0.0549
C48	C1.00	0.623	0.6992	-0.1235
C49	C1.00	0.2816	0.3823	0.0775
C60	C1.00	0.4007	0.7983	0.4532
C62	C1.00	0.4621	0.4056	0.5409
C63	C1.00	0.5605	0.4433	0.513
C64	C1.00	0.412	0.3082	0.5979
C65	C1.00	0.3904	0.5741	0.4818
C66	C1.00	0.299	0.5151	0.4848
C67	C1.00	0.7284	1.0098	0.2653
C68	C1.00	0.3541	0.9479	0.6254
C79	C1.00	0.1457	0.484	0.4807
C81	C1.00	0.5301	0.9518	0.4312
C82	C1.00	0.474	0.9917	0.5165
C83	C1.00	0.6363	1.0198	0.362
C84	C1.00	0.3684	0.7018	0.4582
C85	C1.00	0.4248	0.6673	0.4681
C86	C1.00	-0.043	0.5817	0.4866
C87	C1.00	-0.0641	0.2786	0.5924
C98	C1.00	0.449	0.5386	0.4985
C100	C1.00	-0.0164	0.4502	0.5169
C101	C1.00	-0.0787	0.3539	0.5509
C102	C1.00	-0.0728	0.4833	0.5099
C103	C1.00	0.2427	0.5488	0.4728
C104	C1.00	0.2779	0.6424	0.4592
C105	C1.00	0.3048	0.235	0.6405
C106	C1.00	0.6533	0.5464	0.4337
Cu115	Cu1.00	0.7012	1.3909	0.5442
Cu116	Cu1.00	0.5108	1.1835	0.5506
Cu117	Cu1.00	0.7398	1.2157	0.4789
Cu118	Cu1.00	2.2091	1.7326	0.0348
Cu119	Cu1.00	2.4329	1.7531	-0.04
Cu120	Cu1.00	2.2344	1.5513	-0.0271
H12	H1.00	0.5995	0.1261	0.0557
H13	H1.00	0.6661	0.4986	0.017
H14	H1.00	0.2023	-0.0649	0.394
H15	H1.00	0.1509	-0.1383	0.2731

H16	H1.00	0.2493	-0.0091	0.1289
H17	H1.00	0.6282	-0.0438	-0.1916
H18	H1.00	0.6197	0.0066	0.0028
H19	H1.00	0.656	0.0728	-0.2419
H31	H1.00	0.8081	0.5291	0.0479
H32	H1.00	0.4503	0.2261	0.0424
H33	H1.00	0.9069	0.3196	0.1854
H34	H1.00	1.053	0.3715	0.0291
H35	H1.00	0.9913	0.3593	-0.1028
H36	H1.00	1.0692	0.7439	-0.1148
H37	H1.00	0.9217	0.6537	0.0746
H38	H1.00	0.9955	0.6733	-0.2069
H50	H1.00	0.4238	0.3496	0.0065
H51	H1.00	0.7129	0.279	0.0609
H52	H1.00	0.6163	0.6809	0.0218
H53	H1.00	0.6395	0.7706	-0.1634
H54	H1.00	0.693	0.7029	-0.2593
H55	H1.00	0.2082	0.3741	0.1065
H56	H1.00	0.2554	0.3395	0.23
H57	H1.00	0.3107	0.3544	-0.0446
H69	H1.00	0.3521	0.8203	0.4558
H70	H1.00	0.2705	0.4423	0.499
H71	H1.00	0.7019	0.9451	0.3779
H72	H1.00	0.8035	1.0741	0.2321
H73	H1.00	0.7491	1.0019	0.1132
H74	H1.00	0.3326	0.9934	0.7003
H75	H1.00	0.3005	0.8752	0.7519
H76	H1.00	0.3378	0.9428	0.5073
H88	H1.00	0.1294	0.417	0.4701
H89	H1.00	0.4944	0.7123	0.4686
H90	H1.00	-0.0425	0.5965	0.62
H91	H1.00	-0.1044	0.5873	0.4896
H92	H1.00	0.0407	0.6355	0.3322
H93	H1.00	-0.1359	0.2068	0.637
H94	H1.00	-0.0599	0.2754	0.7271
H95	H1.00	0.0122	0.2943	0.446
H107	H1.00	0.5153	0.585	0.5054

H108	H1.00	0.2339	0.6686	0.4535
H109	H1.00	0.2365	0.2332	0.7764
H110	H1.00	0.2857	0.1625	0.6812
H111	H1.00	0.3114	0.2543	0.4957
H112	H1.00	0.7256	0.5522	0.4039
H113	H1.00	0.6265	0.5753	0.555
H114	H1.00	0.68	0.59	0.2812
N1	N1.00	0.414	-0.1348	0.0119
N2	N1.00	0.3143	-0.1554	0.1048
N4	N1.00	0.4622	0.0854	0.0728
N20	N1.00	1.1039	0.6206	-0.0352
N21	N1.00	1.1044	0.5448	-0.0121
N23	N1.00	0.8606	0.4427	0.0157
N39	N1.00	0.3645	0.5578	-0.0421
N40	N1.00	0.4518	0.641	-0.0918
N42	N1.00	0.5178	0.4835	-0.0013
N58	N1.00	0.5462	1.0791	0.4947
N59	N1.00	0.6445	1.0969	0.401
N61	N1.00	0.4888	0.8567	0.437
N77	N1.00	-0.1649	0.3345	0.5569
N78	N1.00	-0.1622	0.4121	0.5331
N80	N1.00	0.0818	0.507	0.5018
N96	N1.00	0.5647	0.3697	0.5554
N97	N1.00	0.4755	0.2879	0.6066
N99	N1.00	0.417	0.4519	0.5155

Supplementary References

1. Taylor, J. M., Dekura, S., Ikeda, R. & Kitagawa, H. Defect control to enhance proton conductivity in a metal-organic framework. *Chem. Mater.* **27**, 2286–2289 (2015).
2. Celedón, S., *et al.* Second-order NLO active heterotrimetallic schiff base metallopolymer. *J. Inorg. Organomet. Polym. Mater.* **27**, 795–804 (2017).
3. da Silva Montani, S., de Lima, J. F., Zanon Zotin, F. M. & Palacio, L. A. Thermal stability of copper-based MOF under different atmospheres. *J. Therm. Anal. Calorim.* **148**, 119–131 (2023).
4. Shi, X., *et al.* Selective liquid-phase molecular sieving via thin metal-organic framework membranes with topological defects. *Nat. Chem. Eng.* **1**, 483–493 (2024).
5. Lázaro, I. A. A comprehensive thermogravimetric analysis multifaceted method for the exact determination of the composition of multifunctional metal-organic framework materials. *Eur. J. Inorg. Chem.* **2020**, 4284–4294 (2020).
6. Vermoortele, F., *et al.* Synthesis modulation as a tool to increase the catalytic activity of metal-organic frameworks: the unique case of UiO-66(Zr). *J. Am. Chem. Soc.* **135**, 11465–11468 (2013).
7. Wei, R.-J., Zhou, H.-G., Zhang, Z.-Y., Ning, G.-H. & Li, D. Copper (I)-organic frameworks for catalysis: networking metal clusters with dynamic covalent chemistry. *CCS Chem.* **3**, 2045–2053 (2021).
8. Wei, R. J., *et al.* Gold(I)-organic frameworks as catalysts for carboxylation of alkynes with CO_2. *J. Am. Chem. Soc.* **145**, 22720–22727 (2023).
9. Wei, Y.-S., *et al.* A single-crystal open-capsule metal-organic framework. *J. Am. Chem. Soc.* **141**, 7906–7916 (2019).
10. Macrae, C. F., *et al.* Mercury 4.0: from visualization to analysis, design and prediction. *J. Appl. Crystallogr.* **53**, 226–235 (2020).
11. Liu, L., *et al.* Harnessing structural dynamics in a 2D manganese-benzoquinoid framework to dramatically accelerate metal transport in diffusion-limited metal exchange reactions. *J. Am. Chem. Soc.* **140**, 11444–11453 (2018).
12. Wu, Y., *et al.* 2D molecular sheets of hydrogen-bonded organic frameworks for ultrastable sodium-ion storage. *Adv. Mater.* **33**, 2106079 (2021).
13. Yin, S.-J., Zheng, G.-C., Yi, X., Lv, G.-P. & Yang, F.-Q. A metal-organic framework@hydrogen-bonded framework as a matrix for MALDI-TOF-MS analysis of small molecules. *Chem. Commun.* **58**, 6701–6704 (2022).
14. Wang, J., *et al.* In situ assembly of hydrogen-bonded organic framework on metal-organic framework: an effective strategy for constructing core-shell hybrid photocatalyst. *Adv. Sci.* **9**, 2204036 (2022).
15. Reichardt, C. Solvatochromic dyes as solvent polarity indicators. *Chem. Rev.* **94**, 2319–2358 (1994).
16. Huang, R. W., *et al.* Hypersensitive dual-function luminescence switching of a silver-chalcogenolate cluster-based metal-organic framework. *Nat. Chem.* **9**, 689–697 (2017).
