# Supplementary material for: 1D axial heterostructure of hydrogen-bonded framework and metal-organic framework by metalation reaction
Source: Nat Commun. 2025 Nov 5;16:9768. doi: 10.1038/s41467-025-64715-1 (PMC12589647; doi:10.1038/s41467-025-64715-1)
Supplement: Supplementary file 2 — Description of Additonal Supplementary Files [file 41467_2025_64715_MOESM2_ESM.pdf]

Description of Additional Supplementary Files

Supplementary Dataset 1- Cif file of HOF-a
